# Great tits and the city: Distribution of genomic diversity and gene–environment associations along an urbanization gradient

**DOI:** 10.1111/eva.12580

**Published:** 2017-12-20

**Authors:** Charles Perrier, Ana Lozano del Campo, Marta Szulkin, Virginie Demeyrier, Arnaud Gregoire, Anne Charmantier

**Affiliations:** ^1^ Centre d'Ecologie Fonctionnelle et Evolutive, CEFE UMR 5175, Campus CNRS, Université de Montpellier Montpellier Cedex 5 France; ^2^ Wild Urban Evolution and Ecology Laboratory Centre of New Technologies University of Warsaw Warsaw Poland

**Keywords:** genomic, great tit, selection, SNP, urbanization

## Abstract

Urbanization is a growing concern challenging the evolutionary potential of wild populations by reducing genetic diversity and imposing new selection regimes affecting many key fitness traits. However, genomic footprints of urbanization have received little attention so far. Using RAD sequencing, we investigated the genomewide effects of urbanization on neutral and adaptive genomic diversity in 140 adult great tits *Parus major* collected in locations with contrasted urbanization levels (from a natural forest to highly urbanized areas of a city; Montpellier, France). Heterozygosity was slightly lower in the more urbanized sites compared to the more rural ones. Low but significant effect of urbanization on genetic differentiation was found, at the site level but not at the nest level, indicative of the geographic scale of urbanization impact and of the potential for local adaptation despite gene flow. Gene–environment association tests identified numerous SNPs with small association scores to urbanization, distributed across the genome, from which a subset of 97 SNPs explained up to 81% of the variance in urbanization, overall suggesting a polygenic response to selection in the urban environment. These findings open stimulating perspectives for broader applications of high‐resolution genomic tools on other cities and larger sample sizes to investigate the consistency of the effects of urbanization on the spatial distribution of genetic diversity and the polygenic nature of gene–urbanization association.

## 1 INTRODUCTION

Humans substantially modify natural ecosystems, especially since the industrial revolution (Vitousek, Mooney, Lubchenco, & Melillo, 1997). Urbanization, the process by which cities are formed and expand, is one of the major threats to natural ecosystems. Urbanization affects habitats, notably resulting in their loss, modification, or fragmentation (Crooks & Sanjayan, 2006). Urbanization also results in chemical, noise, and light pollution, altered temperatures, novel epidemics, predation risks, all in all resulting in the modification of species assemblage and demography (Aronson et al., 2014; Galbraith, Jones, Beggs, Parry, & Stanley, 2017; Shryock, Marzluff, & Moskal, 2017; Vincze et al., 2017), phenotypic traits (Alberti et al., 2017; Biard et al., 2017; Suárez‐Rodríguez, Montero‐Montoya, & Macías Garcia, 2017), and evolutionary dynamics (Alberti, 2015; Anderies, Katti, & Shochat, 2007; Hendry, Gotanda, & Svensson, 2017). Although urbanization generally results in dramatic local biodiversity declines as a lot of species avoid or are unsuccessful in urban environments, other species are able to cope and even take advantage of such environmental change via plastic or adaptive responses (Lancaster & Rees, 1979; Møller et al., 2012). Understanding the demographic, ecological, and evolutionary processes resulting from urbanization is thus becoming an important goal in conservation and evolutionary biology (Donihue & Lambert, 2014).

Urbanization has various impacts on the demography of species, affecting the density of individuals, their dispersal, their survival, and their reproductive rates, and ultimately affecting genetic diversity and differentiation among populations. In particular, fragmentation and degradation of habitats strongly affect dispersal of individuals and population sizes. Because it leads to reduced dispersal and gene flow, fragmentation caused by urbanization can result in lower genetic diversity and higher neutral genetic differentiation among populations (Gortat et al., 2015; Lourenço, Álvarez, Wang, & Velo‐Antón, 2017; Munshi‐South, Zolnik, & Harris, 2016) as well as increased relatedness among individuals (Chiappero et al., 2011). The impact of these effects of fragmentation on demography and genetic differentiation will, however, depend on the dispersal capacity of species. While fragmentation can enhance genetic differentiation in rodents and amphibians as they are susceptible to terrestrial barriers (Gortat, Rutkowski, Gryczynska‐Siemiatkowska, Kozakiewicz, & Kozakiewicz, 2012; Lourenço et al., 2017), bird species will generally be less affected as they can fly between suitable habitats (Partecke, Gwinner, & Bensch, 2006; but see Björklund, Ruiz, & Senar, 2010). The decrease in habitat quality caused by urbanization may also result in reduced genetic diversity and increased differentiation if population size decreases, thereby increasing the amount of genetic drift (Munshi‐South et al., 2016). Nevertheless, if fragmentation does not impede dispersal, asymmetrical gene flow can occur from more productive populations inhabiting natural habitats into less productive ones inhabiting unfavorable habitats, that is, highly urbanized areas (Björklund et al., 2010). Lastly, habitat choice could also reinforce a neutral genetic differentiation between populations inhabiting natural and urbanized environments. Although habitat choice is known to influence patterns of dispersal, gene flow, genetic differentiation, and evolutionary processes (Dreiss et al., 2012; Edelaar & Bolnick, 2012), it has received limited attention in the case of urbanization studies.

If it imposes divergent selection pressures compared to those acting in wild environments, urbanization may also result in divergence at particular loci underlying adaptive evolution in urbanized environments, detectable through genomic approaches. Many studies have recently investigated genomic footprints of divergent selection that may be implicated in local adaptations, in a wide range of species and contexts (Savolainen, Lascoux, & Merilä, 2013; Tigano & Friesen, 2016). However, these studies were largely restricted to wild populations in natural environments (e.g., well‐known examples in sticklebacks (*Gasterosteus aculeatus*; Hohenlohe et al., 2010), in Atlantic cod (*Gadus morhua*; Nielsen et al., 2009), or in lizards (*Lacerta lepida*; Nunes, Beaumont, Butlin, & Paulo, 2010)). Only a small number of studies have focused on urban genomics, investigating footprints of divergent selection between natural and urbanized environments based on genomewide data. Harris, Munshi‐South, Obergfell, and O'Neill (2012) and Harris and Munshi‐South (2017) investigated genomic shifts in white‐footed mice *Peromyscus leucopus* in New York City, and examined the evolutionary consequences of urbanization. Among the thousands of SNPs screened using genome scans, they identified several candidate genes possibly under positive selection in urban versus rural populations of *P. leucopus*. These outliers were notably involved in metabolic functions and were potentially underlying rapid local adaptation in urbanizing habitats where *P. leucopus* may use different food resources. Such pioneering urban genomic studies have begun to pave the road for investigating genomic footprints of divergent selection between natural and urban wild populations. With the rapid spread of new generation sequencing tools, similar genomewide studies like the one of Harris and Munshi‐South (2017) can be applied to nonmodel organisms, in search of novel candidate genes potentially under selection.

The great tit (*Parus major*) is a small passerine species that has become a model study organism in behavioral ecology and evolutionary biology. Great tits are widespread and abundant across Eurasia and can be found in a wide variety of environments, from natural forests to highly urbanized cities. Previous studies based on microsatellite loci showed relatively small genetic differentiation across Europe, yet higher differentiation among southern populations (Lemoine et al., 2016) as well as within the city of Barcelona (Björklund et al., 2010). Studies comparing the life history and physiology of great tits in forest versus city habitats have recently revealed that, compared to their forest conspecifics, urban great tits lay earlier and smaller clutches, they display faster breath rates and faster exploration scores in a novel environment, have higher levels of neophilia, and their offspring fledge in poorer condition (Bailly et al., 2016; Charmantier, Demeyrier, Lambrechts, Perret, & Gregoire, 2017; Marzluff, 2001; Sprau, Mouchet, & Dingemanse, 2016; Torné‐Noguera, Pagani‐Núñez, & Senar, 2014; Tryjanowski et al., 2016). Given this strong phenotypic divergence and as genomic resources have recently been developed for this species (Laine et al., 2016), the great tit is a good candidate species for urban genomic studies investigating both neutral genetic differentiation between urban and natural populations and potential genomic bases underlying adaptations to urban environments.

Here, we took advantage of the recent advances in RAD sequencing techniques (restriction site‐associated DNA sequencing; see Etter, Bassham, Hohenlohe, Johnson, & Cresko, 2011 and Rowe, Renaut, & Guggisberg, 2011 for introductions about RAD sequencing) and of the availability of the great tit genome to perform a genomewide analysis of individuals breeding across a gradient of urbanization, looking for effects of urbanization on both neutral and adaptive genetic differentiation. Specifically, the first aim of this study was to test the impact of the local level of urbanization on the extent of genomewide genetic diversity and structure in urban great tits. Although small genetic differentiation was expected given recent empirical findings in this species (Laine et al., 2016; Lemoine et al., 2016), we hypothesized that significant population structure and reduction in diversity may occur among populations found in areas with different levels of urbanization (Björklund et al., 2010), potentially resulting from the interplay between reduced dispersal and population size, habitat choice, and local adaptation. The second aim was to search for genomic footprints of divergent selection between sites with different levels of urbanization, and gene–urbanization associations, that may be implicated in adaptation to urban life (Harris et al., 2012).

## 2 METHODS

### 2.1 Study sites, sampling

**Figure 1:**
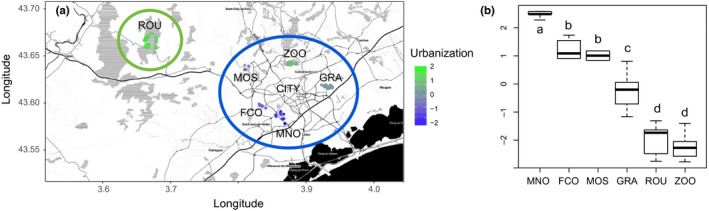
(a) Map of the sampled sites with color scaled urbanization. (b) Boxplots of urbanization scores per site. Correspondences of site abbreviations are found in Table 1

A total of 140 great tits were sampled from 87 nest boxes positioned in five sites with different levels of urbanization in the city of Montpellier (France) and in a rural site: the deciduous forest of La Rouvière (Montarnaud, France) where great tits have been monitored since 1991 (Table 1, Figure 1a). The total number of monitored nest boxes available for great tits was 180 in the city and 70 in the Rouvière forest. The city of Montpellier experienced a high human population expansion since the middle of the 19th century to early 21st century, growing from around 35,000 to 280,000 inhabitants. Six sites are equipped with monitored nest boxes: the zoo of Lunaret (ZOO) which includes a large forested area; Grammont (GRA), Mas Nouguier (MNO), Font‐Colombe (FCO) and Mosson (MOS) which all contain a mixture of urbanized sectors and parks; and La Rouvière (ROU) which is an oak forest located 20 km northwest of Montpellier (Table 1, Figure 1).

**Table 1:** Site name, abbreviation, average nest coordinate per site, average nest urbanization level (see methods), sample size (before and after removing highly related individuals), and observed heterozygosity (Ho). Sites were ordered by average urbanization level. For urbanization level and observed heterozygosity, letters indicate significant differences

Site	Abbreviation	Coordinates (lat. long.)	Urbanization level	Sample size	Ho
Mas‐Nouguier	MNO	43.586 3.862	+2.32^a^	18/18	0.285^a^
Font‐Colombe	FCO	43.597 3.834	+0.87^b^	10/10	0.290^abc^
Mosson	MOS	43.637 3.812	+0.70^b^	5/5	0.290^abc^
Grammont	GRA	43.617 3.932	−0.31^c^	29/26	0.289^ab^
Rouvière	ROU	43.664 3.668	−1.94^d^	47/41	0.294^c^
Zoo	ZOO	43.642 3.878	−2.19^d^	31/25	0.292^bc^

Great tit breeding was monitored in all nest boxes from April to July each year since 1991 in La Rouvière forest and since 2011 in the city of Montpellier. Parents were caught in nest boxes when their nestlings were 10 days old. In 2014, 5–30 μl of blood was sampled from adult breeders for later DNA extraction. Blood was taken from a small neck vein or from a wing vein and stored at 4°C in Queen's buffer (Seutin, White, & Boag, 1991).

### 2.2 Urbanization level

From April to May 2012, vegetation cover (surface covered by oaks, trees and green spaces), and light (artificial night lighting), air and noise (using car traffic to reflect as a proxy) pollutions were recorded within a 50‐m‐radius disk around each nest box (see Demeyrier, Lambrechts, Perret, and Gregoire (2016) for details) which corresponds to the main area prospected by the focal pair within a breeding season (Perrins, 1979). A principal component analysis (PCA) of these variables revealed that they were highly correlated (Demeyrier et al., 2016). Therefore, the first axis of the PCA was chosen as a single continuous variable to reflect the urbanization level at each nest box (Table 1 and Figure 1a), which was thereafter referred to as nest‐level urbanization. Differences in nest‐level urbanization among sites were investigated using an ANOVA and a Tukey's HSD test in R (R Core Team, 2016). Additionally, latitude and longitude were recorded at each nest box (Table 1) with a GPS (Garmin© GSPmap^®^ 62s).

### 2.3 DNA extraction and RAD sequencing

DNA extraction was performed using Qiagen DNeasy Blood & Tissue kits. DNA extraction was randomized across sites. DNA was quantified using a NanoDrop ND8000 spectrophotometer and then a Qubit 2.0 fluorometer with the DNA HS assay kit (Life Technologies). DNA quality was checked on agarose gels. DNA extracts (including two replicates in different lanes to estimate the consistency of the analyses) were sent to MGX (CNRS, Montpellier) for libraries preparation using restriction site‐associated DNA sequencing (RADseq; Baird et al., 2008) with the enzyme SbfI. Each individual was identified using a unique 6‐nucleotide tag. Individual samples were multiplexed in equimolar proportions by groups of 29 or 30 individuals. Individuals were randomized across groups. Each group was sequenced on one of seven lanes of an Illumina HiSeq 2000 (on these lanes were also included juvenile great tit individuals from the same sites but that were not used in this study).

### 2.4 SNP calling and filtering

Raw sequences were inspected with *FastQC* (Andrews, 2010) for quality controls. Reads were treated with *Cutadapt* (Martin, 2011) to remove potential fragments of Illumina adapters. A 10% mismatch was allowed in the adapter sequence. *Stacks 1.32* (Catchen, Hohenlohe, Bassham, Amores, & Cresko, 2013) was used to demultiplex reads, identify RAD loci, and call single nucleotide polymorphisms (SNPs). Reads were filtered for overall quality, demultiplexed, and trimmed to 85 bp using *process_radtags*. One mismatch in the barcode sequence was allowed. BWA‐MEM 0.7.13 (Li & Durbin, 2009) was used to map individual sequences against the reference genome of the Great tit (Laine et al., 2016) using default options. The total assembled contigs spans 1.0 Gb, for an estimated genome size of 1.2 Gb. We used samtools 0.1.19 to build and sort *bam* files (Li et al., 2009). We used *pstacks*, with a minimum stack depth of 5 (m = 5), the SNP model, and α = .05. *cstacks* was used to build the catalogue of loci using *n* = 3. With *sstacks,* loci were searched against the catalog of loci. Finally, individuals were genotyped using the stacks's *populations* module and filtered using *VCFtools* (Danecek et al., 2011). Loci were retained if genotyped in at least 90% of individuals (all individuals from all sites grouped) with individual minimal read depth of 8 (“na” replaced genotypes below a read depth of 8), with a minimum average read depth of 20 across all genotypes, a maximum average read depth of 100 across all genotypes, and a minor allele frequency above 5% (MAF ≥ 0.05) across all individuals (all individuals from all sites grouped). We verified that each individual was genotyped for at least 95% of all loci. We filtered the entire dataset for deviations from Hardy–Weinberg equilibrium at the SNP level (HWE, *p*‐value ≥ .01) in ROU. We did not filter specifically the other populations for HWE due to smaller sample sizes, and we considered that most of the significant deviations in every site would also be found in ROU given the high gene flow among the populations. This HWE filtering step was mainly applied to remove sequencing or SNP calling errors as well as paralogous sequences (Waples, 2015). In turn, this should not interfere with the detection of divergent selection between urban and forest populations as HWE is estimated locally but not across sites. We investigated the average decay of LD in chromosomes 1–20 (the chromosomes on which most of the SNPs (86%) were found), in ZOO (as LD was very similar in all populations), using *gdsfmt* and *SNPRelate* (Zheng et al., 2012). Although the data are not phased, this coarse estimate of LD was useful to evaluate the physical distance between outlier SNPs and neighboring genes that may be relevant for our functional analysis and to discuss the proportion of the genome covered by our RADseq analysis. Finally, we calculated identity‐by‐state IBS among individuals using the R packages *gdsfmt* and *SNPRelate* to (i) estimate consistency of SNP calling on the two replicated individuals, and (ii) identify highly related individuals. One individual was removed for each pair of highly related individuals (i.e., siblings and parent–offspring) to produce a dataset called “*no‐family‐ties*.” The rationale for this procedure is that related individuals could artificially increase *f*_st_ between groups of individuals (see also Szulkin, Gagnaire, Bierne, & Charmantier, 2016).

### 2.5 Investigation of genetic diversity, relatedness, and differentiation

Genetic diversity was inferred through individual observed heterozygosity (Ho) on the *no‐family‐ties* dataset. Ho was estimated using *VCFtools*. Differences in individual Ho were investigated using ANOVAs in R*,* with individuals grouped in the six sites. Finer comparisons of Ho between pairs of sites were achieved using a Tukey's HSD test in R.

Population structure was investigated *via* identity‐by‐state (IBS), fixation index (*f*_st_), a principal component analysis (PCA), and a discriminant principal component analysis (DAPC). We calculated IBS among individuals of the entire dataset to determine whether relatedness among individuals differed between sites and to test whether we could infer dispersal events (via the presence of highly related individuals found in different sites). Differences in IBS between sites were assessed in R using an ANOVA (see also Szulkin et al. (2016) for more details on the method). *f*_st_ were estimated between the six sites using *GenoDive* (Meirmans & van Tienderen, 2004), which takes into account Weir and Cockerham's optimizations of Wright's theoretical index (Wright, 1951) to control for unequal sample sizes (Weir & Cockerham, 1984). The significance of pairwise *f*_st_ values was tested through 1,000 permutations in *GenoDive*. We estimated the *f*_st_ using both the entire dataset and the *no‐family‐ties* dataset. We also estimated a global *f*_st_ (Weir & Cockerham, 1984) between sites for each SNP using *SNPRelate* and represented with a Manhattan plot. The most likely number of genetic cluster was estimated using the function *find.clusters* from the R package *adegenet* (Jombart, 2008) on the *no‐family‐ties* dataset. We used a principal component analysis (PCA) using the function *snpgdsPCA* implemented in the R package *SNPRelate* to depict genetic structure among the sites, using both the entire dataset and the *no‐family‐ties* dataset. Finally, we used a discriminant analysis of principal components (DAPC) implemented in the R package *adegenet* to depict genetic structure among the sites, using the *no‐family‐ties* dataset. We used the cross‐validation procedure from the *adegenet* package using the function *xvalDapc*, to identify the optimal number of principal components, reassigning 30% of the individuals, with a training set based on 70% of the individuals and replicating 100 times the procedure for each set of PCs from 10 to 45 by steps of 5, and then from 5 to 15 by steps of 1.

### 2.6 Effect of geographic distance and of urbanization on genetic diversity, sites differentiation and individual relatedness

First, we estimated the correlations between urbanization and observed heterozygosity, at the site level and at the nest level.

Second, to infer the effect of geographic distance and of urbanization difference on dispersal and genetic differentiation, we inferred correlations between (i) *f*_st_ (estimated on the *no‐family‐ties* dataset) and geographic distance, (ii) *f*_st_ and urbanization difference, (iii) IBS (estimated on entire dataset) and geographic distance, and (iv) IBS and urbanization difference. The correlations of either *f*_st_ or IBS with geographic distance are used to infer potential isolation by distance, whereby dispersal and accumulation of genetic differentiation increase with geographic distance between populations. The correlations of either *f*_st_ or IBS with urbanization difference are used to infer potential habitat choice, whereby dispersal and accumulation of genetic differentiation increase with habitat differences. *f*_st_ is more likely to capture long‐term accumulation of allele frequency differences while IBS captures the present time distribution of relatedness and therefore the dispersal events.

Third, to investigate simultaneously the influence of latitude, longitude, and urbanization on the genetic distance among individuals (using the entire dataset), we used a redundancy analysis (RDA), which is a constrained version of PCA (Legendre & Fortin, 2010; Legendre & Legendre, 2012), implemented in the *Vegan* R package (Oksanen et al., 2007). Using this RDA, we first investigated the portion of the genetic variability that could be explained by a constraining covariance matrix consisting of latitude, longitude, and nest‐level urbanization for each individual. We tested the global significance following 1,000 permutations. Then, we ran marginal effects permutation tests to address the significance of each variable. Ultimately, we focused on the effect of nest‐level urbanization alone, using partial RDA first taking into account the effect of latitude and longitude. Significance was tested running 1,000 permutations. Because the indirect effect of nest‐level urbanization on genetic structure could operate at a scale larger than the 50‐m‐radius sphere around the nests, we performed a second RDA using a site‐specific average level of urbanization (calculated for each site as the average of the nest‐level urbanization values) rather than a nest‐level urbanization.

### 2.7 Search for SNPs, genes, and Gene Ontologies associated with urbanization

We used latent factor mixed model (LFMM; Frichot, Schoville, Bouchard, & Francois, 2013) on the entire dataset to identify SNPs potentially under divergent selection along the urbanization gradient. As urbanization may act as a selection force at different spatial scale on great tits, we implemented three tests using fine‐ to large‐scale incorporation of urbanization. We first used the fine‐scale nest‐level urbanization values (50 m radius from their nest) as explanatory variable (referred as test A). In test B, we used site‐specific average urbanization. In test C, we used a binary test comparing ROU (coded as 0) and the city sites (coded as 1).

These three tests were performed again, excluding the ZOO (referred as tests D, E, F). This was motivated by the fact that birds in the Zoo may experience similar selection pressures as ROU birds (as similar low urbanization values were observed), while being in the perimeter of the city. Therefore, gene flow among urbanized sites from the city and the zoo could limit the response to locally forestlike environment in the zoo and limit the power of outlier tests. Moreover, this site also represented an opportunity to avoid circularity, first identifying outlier loci excluding ZOO and subsequently inspecting by mean of PCAs (detailed next paragraph) whether ZOO individuals were closer to urban or forest birds. ZOO individuals closer to urban birds would suggest restricted effect of selection relatively to gene flow, at small spatial scale, or absence of habitat choice influenced by outlier loci. In contrast, ZOO individuals closer to forest birds would suggest relatively important response to selection at small spatial scale, or of habitat choice influenced by outlier loci.

Five runs (10,000 burn‐in, 100,000 iterations) per LFMM test were used to obtain average z‐scores, representing the strength of the association between a SNP and an explanatory variable, and associated *p*‐values, assuming a unique genetic cluster. We displayed the Z‐scores with Manhattan plots. We estimated *q*‐values from LFMM's *p*‐values, for each of the six tests separately, using the R package *fdrtool* (Strimmer, 2008) and reported the distribution of the results across the six tests (A–F) using (i) Manhattan plots for each test, (ii) histograms of outliers’ *z*‐score distributions for each test, (iii) biplots between tests, and (iv) Venn diagrams (R package *VennDiagram*, Chen & Boutros, 2011; and the Web‐based tool InteractiVenn, Heberle, Meirelles, da Silva, Telles, & Minghim, 2015). On each Manhattan plot was superposed a line representing a kernel‐smoothing moving average using a 10‐Mb‐long window sliding by steps of 10 Kb, to determine whether outlier SNPs were found in genomic areas with average high association with urbanization. We reported a table of the SNPs found in the top 10 of each test, to provide more functional details on the putative genes in which these outliers were found.

We performed PCAs, using the function *snpgdsPCA* implemented in the R package *SNPRelate*, followed by linear models in R, to investigate the proportion of variance in individuals’ scores of urbanization at the nest and at the site explained by the genetic distances between individuals when considering several outlier lists of SNPs with *q*‐values <.05, as well as the entire set of SNPs. This procedure was achieved to inspect the power of gene–urbanization associations. Either the PC1 or PC2 or both were considered to explain individual nest‐ or site‐level urbanization, using a linear model in R. The PCA, realized with the list obtained from tests D, E, and F, was furthermore used to visually inspect the genetic distance of ZOO with other sites.

To assess to which extent genomic associations with urbanizations were polygenic, we inferred, using linear models, the proportion of variance in nest‐level urbanization that was explained by PCAs using an increasing number of SNPs, from 1 to 49,969, sorted by decreasing Z‐score in test A. Although this is a circular examination, it is primarily an attempt to investigate the cumulative nature of the top outlier SNPs from gene–urbanization association tests. We considered only PC1 as this axis captured most of the variation in urbanization while analyzing outliers from test A (as it will be explained in the result section).

Genes were extracted from the great tit reference annotation (NCBI *Parus major* Annotation Release 100). We reported the genes found in the aforementioned lists. Then, gene ontology (GO) enrichment tests, which aim to identify potentially enriched GO terms, were performed in GOrilla (Eden, Navon, Steinfeld, Lipson, & Yakhini, 2009), using as background list of genes the entire set of genes found among the SNPs used in this study (*n* = 5,276). We tested for GO enrichment among genes founds for several lists of outliers (each test separately and several combinations of tests). Enrichment tests were corrected with the FDR method.

## 3 RESULTS

### 3.1 Environmental data

Average urbanization level per site ranged from −2.19 in ZOO to 2.32 in MNO (Table 1, Figure 1a,b). The maximum nest‐level urbanization was 2.9 in MNO, and the minimum was −2.7 in ZOO. The fact that ZOO was found less urbanized than ROU (the forest) is in part due to the absence of motorized vehicles in the zoo, unlike in the forest, as well as its huge vegetation cover. Nest‐level urbanization was significantly different across sites (ANOVA, *F* = 180.3, *p*‐value < 2e‐16). Tukey's HSD test revealed that all pairs of sites had significantly different means in nest‐level urbanization scores (*p* < .05), except FCO and MOS (*p* = .99) and ZOO and ROU (*p* = .52).

### 3.2 SNP calling

Sequencing RAD tags for 140 breeding great tits resulted in a total of 1,815,823,241 raw sequences, with an average of 7,793,233 reads per individual. 107,413 loci were identified in the stacks catalog. After filtering for depth coverage, HWE, and MAF, 49,969 SNPs in 32,756 loci were kept in the final dataset, roughly one locus every 37 Kb. The missing rate per individual ranged from 0.001 to 0.058, with a median of 0.024. The missing rate per locus ranged from 0.000 to 0.100, with a median of 0.014. The average read depth across genotypes (SNP * Individual) was 72 (Appendix S1), and the fifth percentile was 24. Average linkage disequilibrium decreased rapidly within the first Kb between SNPs and then continued to decrease slowly (Appendix S2). The IBSs between each replicated samples were high, respectively, of 0.9999 and 0.9987. Replicates were removed from all subsequent analyses. To construct the *no‐family‐ties* dataset, twelve individuals with IBS superior to 0.80 with other individuals were removed (three in GRA, six in ZOO, and six in ROU).

### 3.3 Genetic diversity, relatedness, and differentiation

There was a general pattern of reduced diversity in individuals from the four most urbanized city sites, in particular MNO, compared to ZOO and to ROU. The observed genomewide observed heterozygosity (Ho) was different among sites (ANOVA: *F* = 11.6, *p*‐value = .000866, Table 1). Ho was lower in MNO (the most urbanized site) than in ROU and in ZOO. Ho was lower in GRA than in ROU.

**Figure 2:**
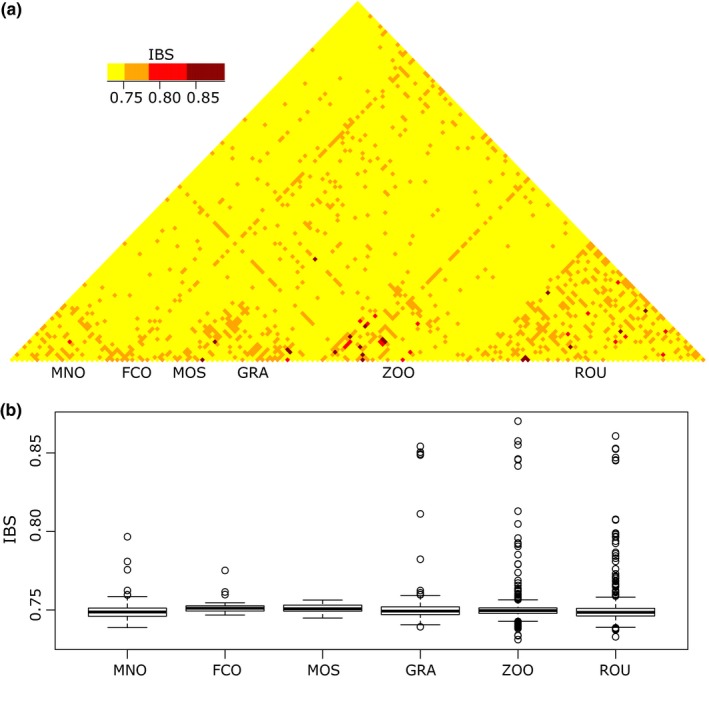
(a) Heatmap of genomic relatedness (IBS) among individuals. (b) Boxplots of IBS among individuals in the six sites. Site abbreviations correspond to Table 1

Pairwise IBS between individuals ranged from 0.73 to 0.89 and was in average of 0.75 (Figure 2a,b). Average IBS values were not significantly different between the sites, using either the entire dataset (ANOVA: *F* = 1.99, *p*‐value = .08) or the *no‐family‐ties*‐dataset (ANOVA: *F* = 1.62, *p*‐value = .15).

**Figure 3:**
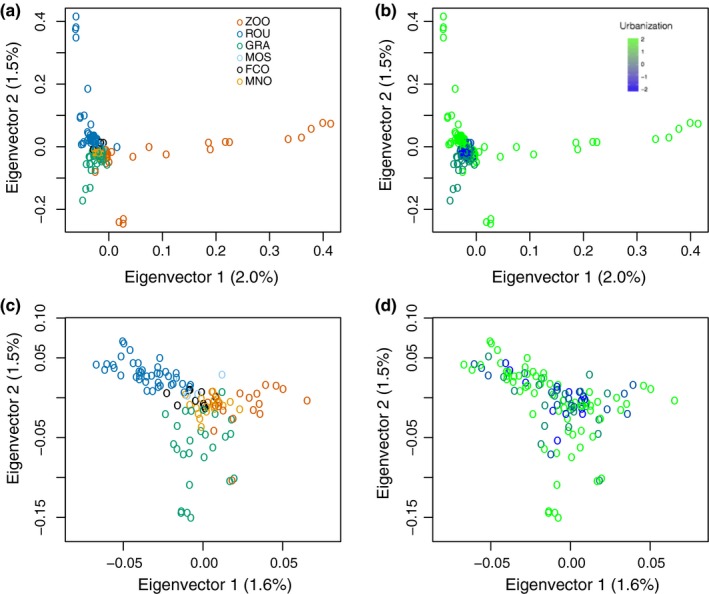
Principal component analyses of individual genotypes based on (a and b) the entire dataset of 49,969 SNPs and all of the 140 individuals, (c and d), the entire dataset of SNPs and *no‐family‐ties* dataset. In (a and c), color code refers to populations. In (b and d), color code refers to urbanization level

**Figure 4:**
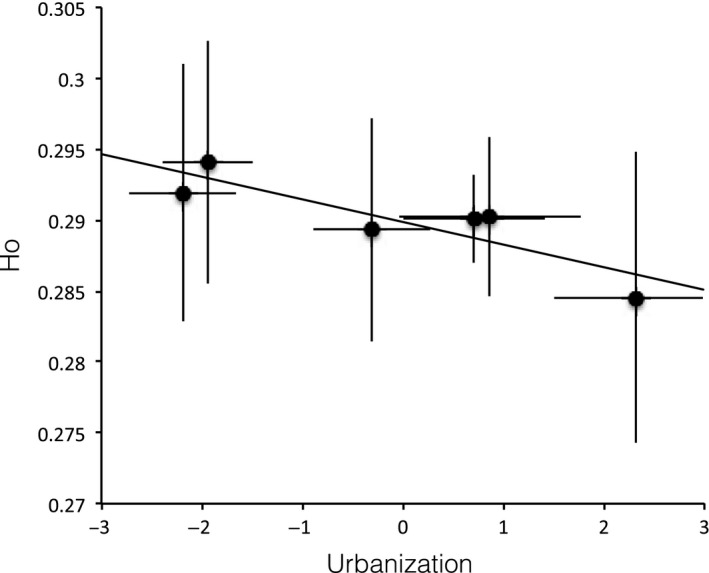
Correlations between site averages of observed heterozygosity and urbanization. Error bars represent standard deviations

The *f*_st_ ranged from 0.004 to 0.009, for an average of 0.007 (Table 2). *f*_st_s were significant for all of the comparisons. The *f*_st_ between the most and least urbanized site was 0.008. When highly related individuals were kept, the genomewide differentiation among the sites ranged from 0.004 to 0.013 for an average of 0.009, illustrating that keeping these individuals could have slightly biased the results. Although average *f*_st_ was very low, there were several SNPs showing relatively high *f*_st_ (Appendix S3a). The *find.clusters* analysis supported the existence of only one genetic cluster. The cross‐validation procedure identified *n* = 10 as the optimal number of principal components of allelic variation to retain for the DAPC (Appendix S4a,b). This use of 10 principal components in the DAPC resulted in average in 82% (median = 87%) of correct individual assignments to their population, revealing a moderate discrimination power of the six groups (Appendix S4b) clearly rejecting the panmixia hypothesis. The DAPC (Appendix S4d) based on 10 principal components and the PCA (Figure 4a–d) both depicted a clear genetic structure between the sites but no particular pattern of differentiation in link with urbanization. The PCA performed better when the no‐family‐tie dataset containing no highly related individuals was used (Figure 3c,d) than when they were included (Figure 3a,b).

**Table 2:** *f*_st_ (upper triangle) and associated *p*‐values (lower triangle) among locations, estimated using the dataset with/without highly related individuals

	MNO	FCO	MOS	GRA	ROU	ZOO
MNO		0.004/0.004	0.009/0.009	0.009/0.008	0.009/0.008	0.012/0.008
FCO	0.001/0.001		0.006/0.006	0.009/0.007	0.007/0.006	0.011/0.006
MOS	0.001/0.001	0.001/0.001		0.010/0.009	0.007/0.006	0.012/0.008
GRA	0.001/0.001	0.001/0.001	0.009/0.001		0.009/0.007	0.012/0.007
ROU	0.001/0.001	0.002/0.001	0.012/0.019	0.001/0.001		0.013/0.008
ZOO	0.001/0.001	0.003/0.001	0.012/0.030	0.001/0.001	0.001/0.001	

### 3.4 Effect of geographic distance and urbanization on genetic diversity and differentiation

At the site level, observed heterozygosity was significantly correlated with urbanization (*r* ^2^ = .76, *p*‐value = .02; Figure 4). At the individual level, observed heterozygosity was also significantly correlated with urbanization but explained much less variation (*r* ^2^ = .09, *p*‐value = .0003).

**Figure 5:**
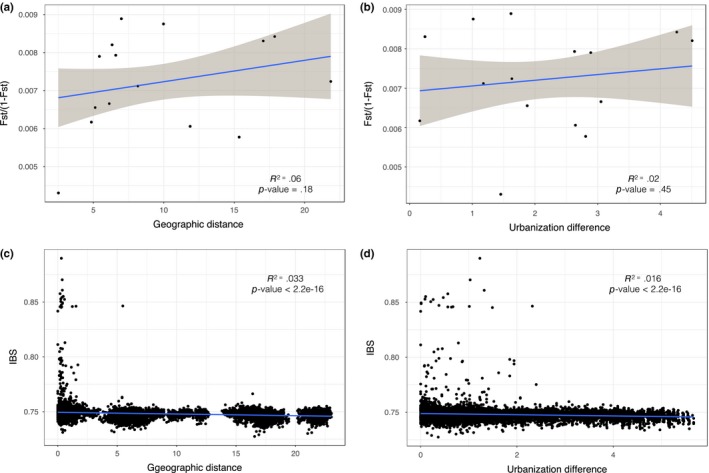
Correlations between (a) genetic differentiation (*f*_st_/(1–*f*_st_)) and geographic distance (Km), (b) genetic differentiation and urbanization differences (absolute value of the difference between local urbanization values), (c) identity‐by‐state and geographic distance, (d) identity‐by‐state and urbanization differences

Genetic differentiation between sites tended (no significant correlation) to increase with geographic distance and with urbanization difference (Figure 5a,b). All, except one, of the few high IBS indices (superior or equal to 0.80, probably corresponding to parent–offspring, full‐sib, or half‐sib relationships) were found within sites, at small geographic distances (Figure 5c). The rest of the IBS indices (below 0.80) only slightly decreased with geographic distance and urbanization difference (Figure 5c,d).

When genetic variance among genotypes was constrained by the full RDA model for latitude, longitude, and nest‐level urbanization, the proportion of variance explained was significant globally (*p* < .001, Table 3), although only the single effect of longitude (*p* < .001) was significant, while nest‐level urbanization and latitude had nonsignificant effects (*p* = .16 and .17, respectively). Using the partial RDA model to test for a nest‐level urbanization effect, after subtracting the effect of longitude and latitude, the proportion of variance explained was not significant (*p* = .21). Urbanization at the nest explained 0.75% of the total genotypic variance (*p* = .20).

**Table 3:** *p*‐Values and variables contributions in the RDA models. Full RDA and partial RDA refer, respectively, to models applied on (i) latitude, longitude, and urbanization as dependent variables, and (ii) urbanization as unique dependent variable with the effects of longitude and latitude removed. Urbanization level was considered either at the nest level or averaged per site. See methods for details. Significant *p*‐values are in bold

	Urbanization level at the nest				Average urbanization level at the site			
	*p*‐Value	RDA axis 1	RDA axis 2	RDA axis 3	*p*‐Value	RDA axis 1	RDA axis 2	RDA axis 3
Full RDA	**.001**	—	—	—	**.001**	—	—	—
% of variance explained	—	0.97%	0.86%	0.72%	—	0.98%	0.87%	0.74%
Latitude	.17	−0.44	0.88	0.19	.093	−0.4	0.88	−0.26
Longitude	**.001**	0.94	−0.34	0	**.001**	0.91	−0.41	−0.02
Urbanization	.157	−0.07	0.97	−0.24	**.013**	−0.02	0.99	−0.05
Partial RDA	.212	—	—	—	**.018**	—	—	—
% of variance explained	—	0.75%	—	—	—	0.78%	—	—
Urbanization	.204	−0.47	—	—	**.014**	−0.31	—	—

When genetic variance among genotypes was constrained by the full RDA model for latitude, longitude, and average urbanization at the site rather than at the nest, the proportion of variance explained was significant globally (*p* < .001, Table 3), and both longitude and average urbanization at the site had significant effects (*p* < .001 and *p* = .01, respectively). After subtracting the effect of longitude and latitude, the proportion of variance explained using the partial RDA model was still significant (*p* = .02). Urbanization average per site explained 0.78% of the total genotypic variance (*p* = .01).

### 3.5 SNPs and genes associated with urbanization and gene ontology enrichment

**Figure 6:**
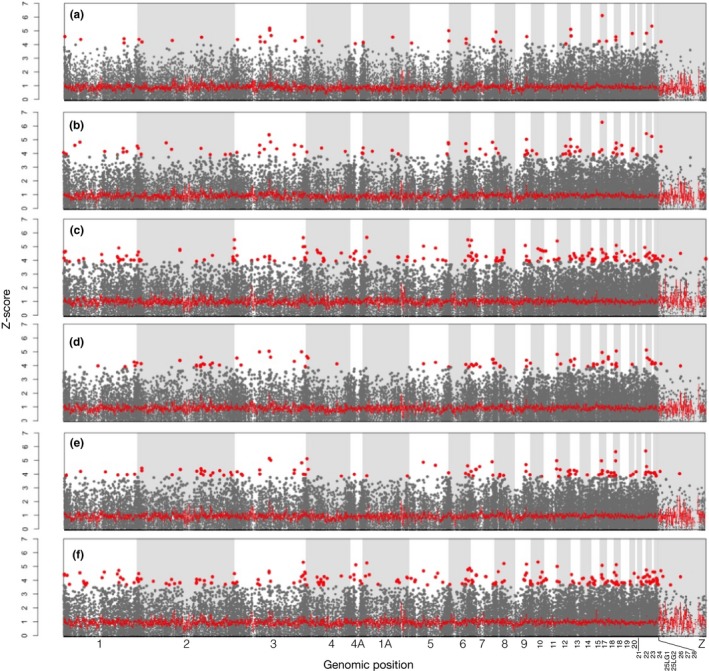
Manhattan plots and sliding window of *z*‐scores of association tests: (a) with urbanization at the nest, (b) with averaged site urbanization, (c) comparing ROU versus the city sites, and d, e, and f showing *z*‐score for similar comparisons as above but excluding ZOO. Red dots correspond to SNPs with *q*‐value <.05 in, at least, the considered test

Several SNPs exhibited relatively high scores of association with urbanization (Figure 6). These association scores were on average decoupled from average *f*_st_ between sites (Appendix S3b,c). Although several hundred outliers were identified, there was little evidence for large *z*‐score peaks that could have been synonymous of strong association at a particular genomic region (but see, e.g., the outlier SNPs in the middle of chromosome 3, Figure 6). The correlations were high between the z‐scores obtained with the quantitative tests at the nest level and at the site level with or without ZOO (*r* ^2^ = .79 between A and B, *r* ^2^ = .79 between D and E, *p* < .001, Appendix S5). The correlations were also relatively high between similar tests made with or without the ZOO (*r* ^2^ AD = .59; *r* ^2^ BE > .58; *r* ^2^ CF = .75, *p* < .001). In turn, the correlations were relatively small between z‐scores values obtained for the quantitative tests (A, B, D and E) and for the binary tests (C and F; average *r* ^2^ = .28, ranging from 0.08 to 0.48, *p* < .001, Appendix S5). At a *q*‐value threshold of 0.05, 45, 89, 514, 81, 114, and 224, SNPs were significant for each of the six tests A, B, C, D, E, and F, respectively (Appendix S6), for a total of 667 SNPs when the six lists were merged. Similar type of tests (quantitative vs. binary) had relatively large proportions of shared outlier SNPs compared to dissimilar tests (Appendix S7). Seven outlier SNPs were found, at *q*‐values <.05 for all of the tests (Appendix S7e). 415 outlier SNPs were only found in one of the six tests.

**Figure 7:**
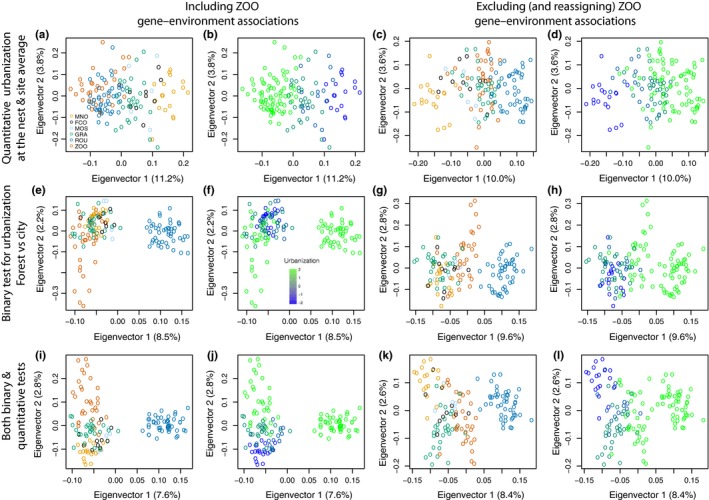
Principal component analyses of individual genotypes based on (a and b): outlier SNPs identified by tests A and B; (d and e): outlier SNPs identified by tests D & E; (e and f): outlier SNPs identified by tests C; (g and h): outlier SNPs identified by tests F; (i and j): outlier SNPs identified by tests A, B and C; (k and l): outlier SNPs identified by tests D, E, and F. In a, c, e, g, i, and k, color code refers to populations. In b, d, f, h, j, and l, color code refers to urbanization level. In tests c, d, g, h, k, and i, ZOO was excluded from gene–environment association tests but was subsequently used for the PCA and representation

**Figure 8:**
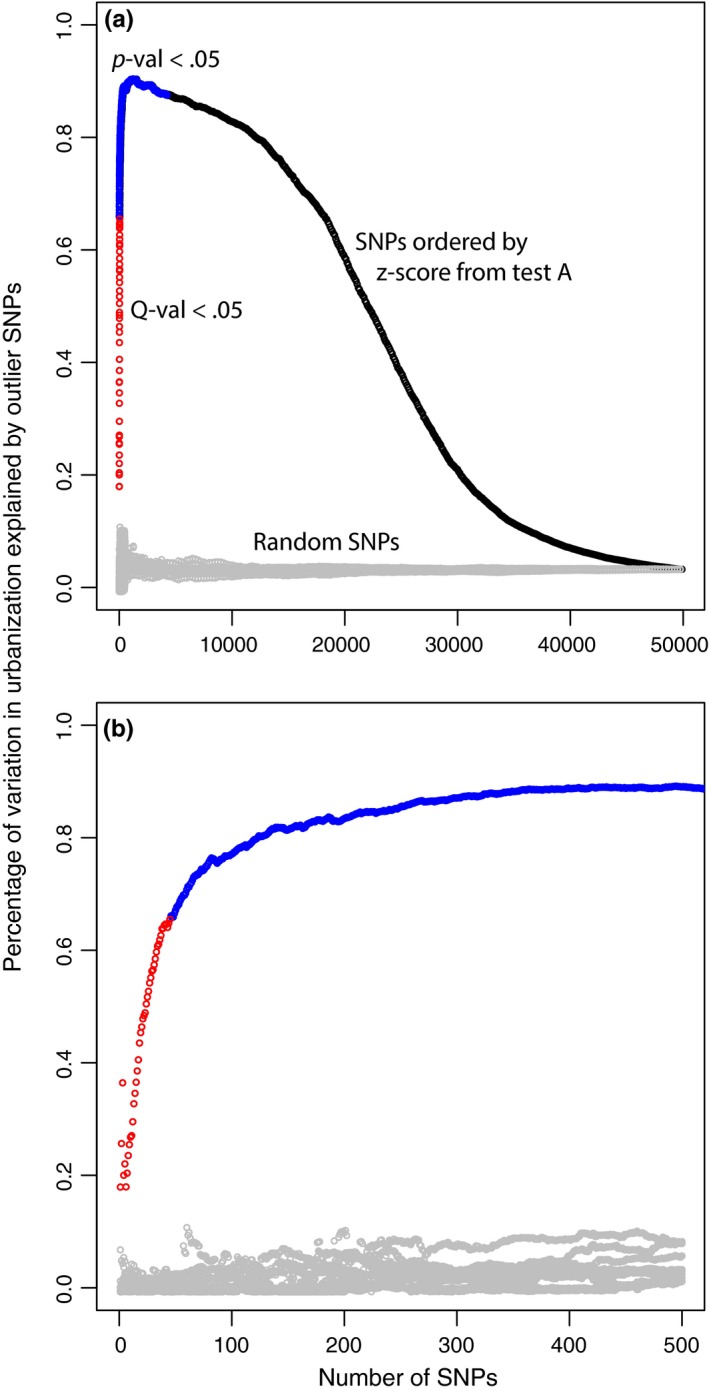
Investigation of putative polygenic gene–urbanization association: a: Percentage of variation in nest‐level urbanization explained by the first principal component of PCAs summarizing genomic variation among individuals for an increasing number of SNPs (from 1 to 49,969), either ordered by decreasing *Z*‐score obtained from the test A (red, blue, and black circles) or randomly added (10 randomizations, gray circles). Models integrating only SNPs with *q*‐values <.05 are shown in red. Models notably integrating SNPs with *q*‐values >.05, but *p*‐values <.05 are shown in blue. Models notably integrating SNPs with *p*‐values >.05 are shown in black. (b) Zoom in for the first 500 SNPs

As expected, the PCAs using outlier SNPs revealed patterns of genetic structure linked to urbanization (Figure 7), in contrast with the PCA using the entire SNP dataset that showed very little genetic structure linked to urbanization (Figure 3b and d) but rather a geographic structure (Figure 3a and c). The first axis of the PCA based on the entire set of SNPs explained 3% of the variation in urbanization score at the nest (Table 4). Taken together, the first and second axes of this PCA explained 6% of the variation in urbanization score at the nest. Similar results were obtained for the site‐level urbanization scores, the first axis of the PCA explained 4% of the variation and 8% were explained by both axes together. In turn, 81% of variance in site‐level urbanization was explained by the first axis of the PCA summarizing interindividual genomic variation at 97 SNPs with *q*‐values <.05 for gene–urbanization tests A & B grouped (Table 4, Figure 7a,b). In contrast, only 15% of variance in urbanization was explained by the first axis of the PCA summarizing interindividual genomic variation at the top 514 SNPs with *q*‐values <.05 for gene–urbanization test C (Table 4, Figure 7e,f), rather depicting ROU versus city differentiation, as expected from the nature of the test C comparing ROU versus city. PC1 and PC2 explained 36% of variation among individuals at these outlier SNPs, with PC2 rather capturing urbanization differences. When combining tests A, B, and C, 71% of variance in urbanization was explained by the two‐first axes of the PCA summarizing interindividual genomic variation at the top 599 SNPs with *q*‐values <.05 (Table 4, Figure 7i,j), PC1 rather capturing ROU versus city differentiation, and PC2 capturing differentiation along the urbanization gradient. When the ZOO was removed from outlier tests but reintegrated for PCAs, ZOO genotypes had intermediate PC1 and PC2 scores between ROU and city sites. The proportion of variance in urbanization at the nest explained by the first axis of PCAs summarizing interindividual genotypic distances increased sharply from 18% to 90% (Figure 8a) with the increase in the number of SNPs added to the model, by decreasing *Z*‐score from test A. The increase in *r* ^2^ was particularly sharp, with the 45 first SNPs (*q*‐values <.05, Figure 8b) explaining 66% of the variation in urbanization at the nest, 80% being reached with 123 SNPs and 85% with 240 SNPs. After a plateau, the proportion of variance explained in urbanization decreased down to 3% explained with the entire dataset of SNPs (Figure 8a). In contrast, there was little effect of the number of SNPs on the percentage of variation explained when SNPs were added randomly to the PCAs (Figure 8a,b, gray circles).

**Table 4:**
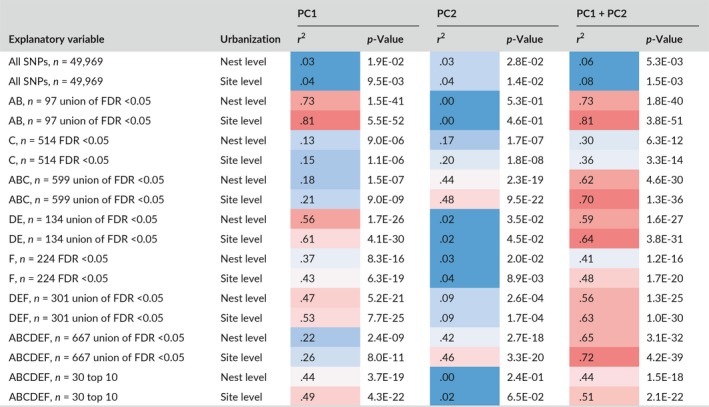
Variation in urbanization score at the nest and at the site explained by the two‐first PCA axes summarizing the genomic variation among individuals, using all the SNPs or different outlier subsets from gene–urbanization associations. *r* ^2^ are color coded using a blue‐red gradient

SNPs from the entire dataset were found in 5276 genes. 266 genes were identified among the 661 SNPs found at least once with a *q*‐value <.05 (Appendix S6). None of the seven SNPs below 0.05 *q*‐values at the intersection of the six tests were found in genes. 12 genes were identified among the 30 SNPs found in the top 10 *z*‐scores of each test (Table 5; Appendix S8). Performing GO tests for genes found for different sets of SNPs with *q*‐values <.05, we detected 42 enriched GO terms (*p*‐val < .0001) compared to the background list of the 5276 genes (Table 6). However, none of the GO were significantly enriched after FDR correction.

**Table 5:** The 30 SNPs and corresponding functional annotations found at the union of the top 10 SNPs of each of the six LFMM test. SNPs are ordered by genomic position. “na” means that the SNP was in an intergenic region

Chrom	Position	*n* outlier tests	A	B	C	D	E	F	Gene abbreviation	Gene name
1	25541327	1		x					CUL4A	Cullin 4A
1A	6049393	2			x	x			FRMD4A	FERM Domain Containing 4A
3	711321	1			x				XRN2	5′‐3′ Exoribonuclease 2
3	39324763	1					x		RPS6KA2	Ribosomal Protein S6 Kinase A2
3	54412965	4	x	x			x	x	na	na
3	54412968	4	x	x			x	x	na	na
3	54413011	4	x	x			x	x	na	na
3	56699164	2		x				x	na	na
3	104563224	1					x		na	na
3	107202050	2			x	x			na	na
4	1271239	2					x	x	na	na
4A	9107560	1				x			SLC25A43	Solute Carrier Family 25 Member 43
5	22593135	1			x				na	na
5	60966129	1	x						FANCM	Fanconi Anemia Complementation Group M
6	29555585	1			x				CPXM2	Carboxypeptidase X, M14 Family Member 2
6	32208338	2			x	x			TCERG1L	Transcription Elongation Regulator 1 Like
7	1817393	1			x				ACKR3	Atypical Chemokine Receptor 3
8	1800681	1	x						na	na
8	14554424	1				x			LOC107208051	na
9	16210793	1			x				PRSS56	Protease, Serine 56
9	18034856	2		x		x			na	na
10	11334760	1				x			na	na
12	487655	4			x	x	x	x	na	na
13	573280	2	x	x					na	na
17	3241094	5	x	x		x	x	x	na	na
19	3617547	4			x	x	x	x	CUX1	Cut Like Homeobox 1
19	3617556	1						x	CUX1	Cut Like Homeobox 1
21	3689856	1	x						CAMTA1	Calmodulin Binding Transcription Activator 1
27	390659	2	x	x					na	na
25LG1	437844	4	x	x			x	x	na	na

**Table 6:** Enriched GO terms for the SNPs with significant *q*‐values for several gene–urbanization association tests and several tests combinations

GO term	Description	*p*‐Value	*q*‐value	A	B	AB	C	D	E	F	DE	ABDE	CF	ABCDEF
GO:0051270	Regulation of cellular component movement	4.68E‐05	2.67E‐01				x	x					x	x
GO:0040012	Regulation of locomotion	2.96E‐04	6.73E‐01				x	x					x	x
GO:0007411	Axon guidance	9.69E‐04	1.00E+00				x						x	x
GO:0032879	Regulation of localization	6.71E‐05	2.55E‐01				x						x	x
GO:0051239	Regulation of multicellular organismal process	1.55E‐04	4.41E‐01				x						x	x
GO:0071625	Vocalization behavior	3.39E‐05	3.86E‐01				x						x	x
GO:0005891	Voltage‐gated calcium channel complex	3.35E‐04	4.84E‐01				x						x	x
GO:0051960	Regulation of nervous system development	5.51E‐04	8.97E‐01											x
GO:0050767	Regulation of neurogenesis	8.43E‐04	1.00E+00											x
GO:0060013	Righting reflex	4.92E‐04	9.34E‐01											x
GO:0005245	Voltage‐gated calcium channel activity	4.49E‐04	1.00E+00											x
GO:0040019	Positive regulation of embryonic development	2.75E‐04	1.00E+00		x	x			x	x	x	x		
GO:0010470	Regulation of gastrulation	1.19E‐04	6.79E‐01							x	x	x		
GO:0051271	Negative regulation of cellular component movement	4.32E‐04	1.00E+00									x		
GO:0040013	Negative regulation of locomotion	4.32E‐04	1.00E+00									x		
GO:0034063	Stress granule assembly	5.81E‐04	1.00E+00									x		
GO:0048856	Anatomical structure development	4.38E‐04	8.32E‐01				x						x	
GO:0034702	Ion channel complex	6.23E‐04	4.51E‐01				x						x	
GO:0032501	Multicellular organismal process	1.94E‐04	5.53E‐01				x						x	
GO:0097485	Neuron projection guidance	8.59E‐04	8.15E‐01				x						x	
GO:0043506	Regulation of JUN kinase activity	6.93E‐04	9.87E‐01				x						x	
GO:0044057	Regulation of system process	8.56E‐04	8.87E‐01				x						x	
GO:0001964	Startle response	7.57E‐04	8.62E‐01				x						x	
GO:0051861	Glycolipid binding	5.16E‐04	7.68E‐01		x	x			x		x			
GO:2000543	Positive regulation of gastrulation	3.11E‐04	1.00E+00		x	x								
GO:0006477	protein sulfation	3.11E‐04	1.00E+00		x	x								
GO:0051923	Sulfation	5.16E‐04	1.00E+00		x	x								
GO:0015015	Heparan sulfate proteoglycan biosynthetic process, enzymatic modification	3.11E‐04	8.87E‐01			x								
GO:0008146	Sulfotransferase activity	3.80E‐04	1.00E+00			x								
GO:0016782	Transferase activity, transferring sulfur‐containing groups	7.48E‐04	7.42E‐01			x								
GO:0022011	Myelination in peripheral nervous system	5.48E‐04	1.00E+00						x		x			
GO:0032292	Peripheral nervous system axon ensheathment	5.48E‐04	1.00E+00						x		x			
GO:0003143	Embryonic heart tube morphogenesis	5.66E‐04	1.00E+00							x				
GO:0001947	Heart looping	3.85E‐04	1.00E+00							x				
GO:0042552	Myelination	7.99E‐04	1.00E+00							x				
GO:0045995	Regulation of embryonic development	4.59E‐04	1.00E+00							x				
GO:1900087	Positive regulation of G1/S transition of mitotic cell cycle	5.84E‐04	1.00E+00	x										
GO:0010769	Regulation of cell morphogenesis involved in differentiation	8.94E‐04	1.00E+00		x									
GO:0002686	Negative regulation of leukocyte migration	8.03E‐04	1.00E+00					x						
GO:0045663	Positive regulation of myoblast differentiation	8.03E‐04	1.00E+00					x						
GO:0030334	Regulation of cell migration	2.54E‐04	1.00E+00					x						
GO:2000145	Regulation of cell motility	3.39E‐04	1.00E+00					x						

## 4 DISCUSSION

Using RAD sequencing, we investigated (i) the potential effect of urbanization on genetic diversity and differentiation in great tits, and (ii) the potential existence of genomic footprints of divergent selection driven by urbanization. Observed heterozygosity was only slightly, yet significantly, lower in the most urbanized sites compared to the least urbanized ones. Furthermore, a small but significant proportion of genetic variance was explained by urbanization. These results either suggest that gene flow was only slightly limited along this urbanization gradient or alternatively that relatively large effective population sizes and relatively recent urbanization slow down the rise of genetic differentiation. Although urbanization explained a small proportion of genetic variance, such deviation from panmixia may be sufficient to allow for the rise of local adaptation (Lenormand, 2002). This result is furthermore in line with similarly small genetic structure between habitats found elsewhere in studies on small‐scale local adaptation in blue tits *Cyanistes caeruleus* (Charmantier, Doutrelant, Dubuc‐Messier, Fargevieille, & Szulkin, 2016; Szulkin et al., 2016). Our search for SNP–urbanization associations revealed several gene–urbanization associations across the genome, suggesting little evidence for an oligogenic but rather a polygenic response to selection. A top subset of 97 SNPs associated with urbanization explained 81% of the variance in urbanization score. A polygenic response to urbanization may be concordant with both the small genomewide differentiation along the urbanization gradient, the relatively recent rise of urbanization, and the expectations that several selective agents may act in urban environments on several complex fitness traits. We discuss below the implications of these results for the evolutionary potential and local adaptation of great tit urban populations and the need for further analyses of genomewide patterns of differentiation linked to urbanization, notably including more replicates from other cities, using both larger genomic coverage and ample size, and integrated in a polygenic analytical framework.

### 4.1 Reduced heterozygosity in urban birds

Genomic data make it possible to measure genetic diversity with great precision. In this study, RADseq exposed that individual heterozygosity was significantly slightly lower in the more urbanized sites than in the less urbanized ones (Figure 4). This result is in line with the reduced genetic diversity observed in urban blackbird (*Turdus merula*) populations compared to rural ones (Evans et al., 2009). Similarly, large increases in relatedness and decreases in genetic diversity have been documented in urban populations for less mobile taxa like rodents (C*alomys musculinus*; Chiappero et al., 2011), foxes (*Vulpes vulpes*; Wandeler, Funk, Largiader, Gloor, & Breitenmoser, 2003), or salamanders *(Desmognathus fuscus*; Munshi‐South, Zak, & Pehek, 2013 *; Salamandra salamandra*; Lourenço et al., 2017). Such patterns suggest that population sizes are slightly smaller and/or less connected in more urbanized sites, possibly in relation to various environmental constrains, including lower resource quality and availability, chemical, light and noise pollution, and anthropogenic disturbance (Dubiec, 2011; Hedblom & Söderström, 2011; Koivula, Kanerva, Salminen, Nikinmaa, & Eeva, 2011; Longcore, 2010). However, in this study, heterozygosity was only slightly different across the urbanization gradient. This may be congruent with the small *f*_st_ found among populations, either suggesting the presence of gene flow or a lag from demographic to genetic effects in a context of relatively recent urbanization. Admittedly, having only six‐point estimates of heterozygosity is rather scarce, and genotyping more sites and possibly in different cities is required for generalization of this pattern.

Given the implications of heterozygosity for individual fitness and population evolutionary potential, slightly reduced heterozygosity detected in urban areas may have important consequences for the adaptive potential of urban great tit populations. Several theoretical and empirical studies showed negative effects on fitness resulting from reduced heterozygosity and sometimes associated inbreeding (Crnokrak & Roff, 1999; Reed & Frankham, 2003; Theodorou & Couvet, 2006). Moreover, reduced diversity impedes adaptive response to stressful conditions (Bijlsma & Loeschcke, 2012). Recent analyses of life‐history traits in the focal populations of great tits have revealed that urban great tits in Montpellier lay smaller clutches and have lower hatching success than their conspecifics breeding in the forest ROU (Charmantier et al., 2017). Surprisingly, however, this study showed that rural and forest birds did not differ in fledging success. Future efforts will need to integrate survival analyses in the comparison of fitness between rural and forest birds. The slight decreased heterozygosity may therefore have implications for the potential of adaptation of great tit populations in urban environments, although whether such reduced diversity in urban environments is generalizable and associated with demographic and/or selective processes remains to be examined.

### 4.2 Low but significant neutral genetic differentiation along the urbanization gradient

The overall low but significant genetic differentiation between sites with different urbanization levels suggests a relatively small, although significant, effect of urbanization on great tit genetic structure along a rural/urban gradient. Interestingly, the genetic structure revealed here is weaker than the *f*_st_ estimated in two studies investigating great tits in urban versus rural areas. First, Lemoine et al. (2016) found an *f*_st_ of 0.018 between Montpellier City and La Rouvière Forest. Second, *f*_st_ among close sites in Barcelona City ranged from 0.018 to 0.19 (Björklund et al., 2010). Such higher differentiations found in these previous studies might, however, be explained by the use of highly polymorphic microsatellite genetic markers and relatively small sample sizes. In the Barcelona study (Björklund et al., 2010), it is also possible that the structure of the city presents a case of extremely reduced patch sizes and population sizes together with high fragmentation due to urbanization, leading to increased differentiation. From a broader perspective, our results also contrast with other vertebrate studies showing a stronger impact of urbanization on the genetic structure of species with lower dispersal capacities (Munshi‐South & Kharchenko, 2010; Munshi‐South et al., 2013; Wandeler et al., 2003).

In turn, although low, the significant genetic variance explained by urbanization may suggest that genetic differentiation due to urbanization is on the rise. The process of differentiation could in fact be partly slowed by large effective population size and the relatively recent rise of urbanization. This hypothesis is sustained by the relatively good definition of populations based on IBS and the almost absence of highly related individuals in different sites, suggesting little recent gene flow among sites. Furthermore, the small but significant genetic differentiation between groups of great tits that are separated by a maximum of 17 km (*f*_st_ of maximum 0.009) found in this study matches the *f*_st_ of 0.01 observed earlier, using a SNP chip, between two much more geographically distant populations, respectively, from the Netherlands and the United Kingdom (Van Bers et al., 2012). It is also similar to the *f*_st_ of 0.012 found among several distant sites across Europe using SNP from genome resequencing (Laine et al., 2016), and to the small genetic differentiation found across European populations of great tits by Lemoine et al. (2016) using microsatellite markers. These studies tend to highlight that despite small average dispersal distances (e.g., mean great tit natal dispersal distance in males/females were 498 m/643 m in Dingemanse, Both, Van Noordwijk, Rutten, & Drent, 2003; and 528 m/788 m in Szulkin & Sheldon, 2008), genetic structure remains weak, potentially resulting from the effect of reduced genetic drift in large populations limiting genetic differentiation. Although large gene flow might limit the potential for local adaptation (Lenormand, 2002), the second hypothesis of rather small gene flow coupled with small genetic drift in large populations is compatible with local adaptation processes. On a practical note, relatively low average genomewide differentiation facilitates the detection of relatively weak footprints of selection and gene–environment associations.

### 4.3 Gene–urbanization associations

This is the first study, to our knowledge, to achieve genomewide SNP scans searching for specific associations between SNPs and urbanization level in a passerine bird. Our results of an absence of SNPs strongly associated with urbanization, but evidence for numerous small gene–urbanization associations does not support the hypothesis that urbanization could provoke a strong response to selection in one or a few oligogenic traits (but see the following paragraph for an alternative hypothesis). The absence of strong gene–urbanization associations may be due to relatively large gene flow, suggested by low *f*_st_, compared to the strength of selection. This could also suggest that divergent selection could be relatively recent between Montpellier city and neighboring forests and that more time is needed, especially in the face of large effective population sizes, to see large effects of selection on individual loci. For example, it has been shown that urban populations of species that have a longer history of inhabiting urban areas have lower fear of humans, suggesting relatively slow local selection or acclimation for reduced responsiveness to humans in urban areas (Symonds et al., 2016). Another alternative is a power issue caused by a low genome coverage (i.e., missing linkage blocks, Lowry et al., 2016; but see McKinney, Larson, Seeb, & Seeb, 2016 & Catchen et al., 2017). This hypothesis needs to be considered carefully as we found rapid decay in average linkage disequilibrium (as also shown in this species by Bosse et al., 2017). Furthermore, fewer loci of larger effects and tighter linkage may resist to the homogenizing effect of gene flow and participate to local adaptation in heterogeneous environments connected with high gene flow (Lenormand, 2002; Yeaman & Whitlock, 2011), hence potentially decreasing the chance of finding them with modest coverage and small linkage disequilibrium. Lastly, our modest sampling (140 individuals) may also lack power to unravel relatively small gene–urbanization associations in a context of relatively high homogenizing effect of gene flow.

In turn, our results of numerous small gene–urbanization associations explaining a large proportion of variance in urbanization score (81% explained by a subset of 97 SNPs) may be in line with the hypothesis that selection regimes resulting from urbanization diverge from natural environments in many aspects and act on several potentially complex traits. A formal comparison of the force, shape, and direction of natural selection across the urbanization gradient would be pivotal in understanding how divergent selection really acts. The theory of quantitative genetics and the recent advances in sequencing and quantitative genomics show that variation in adaptive traits often has polygenic origins (Pritchard, Pickrell, & Coop, 2010; Purcell et al., 2009). In the particular context of high gene flow, and for traits that are genetically highly redundant, local adaptation may occur via rapid small frequency shifts at many alleles of small effects that are prone to swamping in the face of gene flow (Yeaman, 2015). In the great tit, the recent use of a large SNP array (650,000 SNPs) on many (2000) individuals to elucidate the architecture of a heritable trait, laying date, in a wild population, shows no large effect locus (Gienapp, Laine, Mateman, Van Oers, & Visser, 2017). Similarly, Bosse et al. (2017) searched for gene associations with bill length in nearly 1000 birds and found that 3009 SNPs contributed to bill length variation. These studies illustrated that these traits were highly polygenic in this species. In such cases, increasing genomic coverage and sample size, and applying individual SNP genomewide associations and genome scans may not be sufficient to understand the genetic architecture of complex traits’ variation. In such cases of complex traits’ variation and polygenic adaptation with a mixture of small‐to‐moderate effects size genes, it will be important to consider polygenic scoring (Berg & Coop, 2014; Euesden, Lewis, & O'Reilly, 2014), gene sets analyses (Daub et al., 2013; Gouy, Daub, & Excoffier, 2017), and regional chromosome partitioning (Gienapp, Fior et al., 2017; Robinson, Santure, & DeCauwer, 2013; Santure et al., 2013) as promising tools to identify sets of markers, sets of genes, and genomic regions of interest. It will also be essential to maximize both the number of SNPs and the number of individuals to confidently detect the contributions of small effect variants (Robinson, Wray, & Visscher, 2014; Zhou & Stephens, 2012).

From a functional perspective, we can compare our results to two transcriptomic studies, which identified differentially expressed genes between rural and urban white‐footed mice (*Peromyscus leucopus*; Harris et al., 2012) and urban great tits (Watson, Videvall, Andersson, & Isaksson, 2017) and to one study of footprints of selection in urban white‐footed mice (*Peromyscus leucopus*; Harris & Munshi‐South, 2017). Almost no GO terms or genes were found in common between our analysis and these former studies. These studies reported expression differences in genes related to immune system and to metabolic processes. In contrast, our study suggested that several of the enriched GO terms were related to neural functions, which could be in line with the personality traits differences found between urban and rural birds, including in the focal population (Atwell et al., 2012; Charmantier et al., 2017; Minias, 2015; Miranda, Schielzeth, Sonntag, & Partecke, 2013). This may also be congruent with the overrepresentation of genes related to neuronal functions among regions under selection in the great tit genome at a much larger geographic scale (Laine et al., 2016). Taken altogether, these results suggest that selection acting on this species both during its long‐term and short‐term evolution targeted similar important biological functions related to the neural functions. Nevertheless, caution should be taken while interpreting these results, especially as none of the GO was significantly enriched after FDR correction.

### 4.4 Which relevant spatial scale for studying evolutionary effects of urbanization?

The spatial scale at which urbanization impacts wild populations is an important consideration for studies investigating the effects of urbanization on evolutionary trajectories of wild populations. The results of both the RDA and the gene–urbanization associations may educate us on the relevant spatial scale to examine the impact of urbanization in the study system. The fact that the RDA showed a significant effect of urbanization when using a site‐average urbanization but not with a nest‐level urbanization suggests that the spatial scale at which urbanization acts on great tits is larger than the immediate 50 m radius around the nest and may rather integrate several hundred meters around the nest. This is congruent with the fact that (i) great tits usually explore and forage in a relatively vast area around the nest (ca. 3,500–4,000 m^2^ according to Naef‐Daenzer, 2000), and that (ii) the individuals breeding in a given nest may have dispersed from a relatively close nest (see natal dispersal distances provided in the above section). Therefore, while nest‐level urbanization might be related to annual urbanization pressure, site‐level urbanization may be more representative of the level of urbanization individuals encounter during their lifetime. Similarly, the fact that the several gene–environment association tests yielded different SNPs, genes, and enriched GOs, may also reveal that urbanization acts at a specific spatial scale as a selective pressure and that association tests capture different genomic regions and/or have different power. The nest‐value urbanization, used as exploratory variable in the association tests A and D, was a precise description of urbanization at the nest level at the time of breeding. Despite its precision, it may not be entirely representative of the urbanization pressure exercised on individuals’ during their entire lives, depending on individuals’ dispersal and movements. Conversely, comparing birds from the forest to birds from the four most urbanized sites, in the association tests C and F, used a binary investigation of the effect of urbanization at the gene pool level. Such a test is more likely to maximize the detection of the biggest genomic gene–urbanization associations at longer time span and larger geographic scale, but may at the same time neglect more fine‐scale variations in urbanization and may be influenced by isolation by distance. Based on genomic and environmental data only, it is difficult to resolve which of the results of the tests conducted from fine‐ to large‐scale urbanization assessment make more sense and we propose that it may be interesting to combine the results of these several tests, taking advantage of environmental gradients to conduct such a strategy.

## 5 CONCLUSION AND PERSPECTIVES

For the first time in a passerine bird, this study shows a small yet significant effect of urbanization on genomewide diversity and differentiation. This result contrasts with the relatively high effects of urbanization on genetic diversity and differentiation observed for terrestrial animals with lower dispersal capacities compared to birds (e.g., *Peromyscus* spp.; Munshi‐South et al., 2016) but also with the results obtained previously for great tits in Barcelona City (Björklund et al., 2010). Nevertheless, the results of a significant slight decrease in urban birds’ heterozygosity may have implications for the adaptive potential of great tit populations in urban environments. Furthermore, the small but significant genetic variance explained by urbanization may be indicative that gene flow is slightly reduced along the urbanization gradient, potentially allowing for local adaptation to occur (Lenormand, 2002). This context of small genomewide differentiation may furthermore be favorable to the identification of the genomic footprints of divergent selection between urban and rural environments as little confounding effect of spatial and historical structure is expected. Accordingly, we identified numerous genomic regions most likely to be associated with differences in urbanization level and explaining a large part of the variation in urbanization score, possibly suggesting polygenic response to urbanization.

Several research avenues may be of interest for a generalization but also a finer understanding of the neutral and selective genetic effects of urbanization. First, long‐term monitoring of great tits in Montpellier and surroundings should allow in a few years to run capture–mark–recapture models to better estimate demographic parameters such as population size, as well as apprehend dispersal patterns across the different urban areas. Second, studying multiple pairs of urban and rural populations and other urban gradients would attest the robustness of our results, but also determine whether initial groups of urban‐adapted individuals sequentially colonized multiple urban areas or if independent colonization and subsequent selection occurred (Evans, Hatchwell, Parnell, & Gaston, 2010). As urbanization is a recent selective pressure, colonization and subsequent response to selection will most likely be independent. It would therefore be interesting to compare the genes under divergent selection in replicated urban environments. For example, Mueller, Partecke, Hatchwell, Gaston, and Evans (2013) showed moderate parallelism in variations at the SERT gene in 12 pairs of urban and rural blackbird populations. Third, regarding the geographic scale at which studying urbanization effects, spatially explicit simulations of genetic data considering different urban landscapes and population features could help in determining the spatial resolution at which measuring urban explanatory variables. Fourth, increasing genomic resolution (i.e., genotyping more SNPs) could help in discovering more genomic variants potentially implicated in adaptation to urban environments (Lowry et al., 2016; but see McKinney et al., 2016 & Catchen et al., 2017). This could be done by increasing the number of sites targeted by restriction enzymes in a new RADseq study or via whole‐genome resequencing, similarly to the study of Laine et al., 2016 at a larger geographic scale. Fifth, in a context of large gene flow and polygenic architecture of complex traits and adaptation, it will be important to increase the number of individuals to detect infinitesimal signals (Robinson et al., 2014; Zhou & Stephens, 2012). Lastly, statistical approaches to consider polygenic signals, by estimating polygenic scores of adaptation (Berg & Coop, 2014; Gagnaire & Gaggiotti, 2016; Stephan, 2016), identifying gene sets (Daub et al., 2013; Gouy et al., 2017), and partitioning additive variance throughout the genome (Robinson et al., 2013; Santure et al., 2013) will probably be of high interest in this context of polygenic adaptation. These research aims are likely to be tested in the near future thanks to an increasing interest in recent years in both polygenic adaptation (Berg & Coop, 2014; Boyle, Li, & Pritchard, 2017; Pritchard et al., 2010; Purcell et al., 2009; Yeaman, 2015) and urban ecology research (Alberti et al., 2017; Hendry et al., 2017; Ibáñez‐Álamo, Rubio, & Bitrus Zira, 2017; Johnson & Munshi‐South, 2017).

## DATA ACCESSIBILITY

Data available from the Dryad Digital Repository: https://doi.org/10.5061/dryad.60p50: (i) individual genotypes in a VCF file, (ii) a text file with site, geographic coordinates, and urbanization scores at the nest and site, for each individual.

## AUTHORS’ CONTRIBUTIONS

AG, MS, AC, and CP designed the study; VD, AG, AC, and CP contributed to fieldwork; CP did the bioinformatics; CP and ALDC performed data analysis; CP wrote the paper; all authors contributed to improve the manuscript.
