# Supplementary figures and images for: Great tits and the city: Distribution of genomic diversity and gene–environment associations along an urbanization gradient
Source: Evol Appl. 2017 Dec 20;11(5):593–613. doi: 10.1111/eva.12580 (PMC5979639; doi:10.1111/eva.12580)

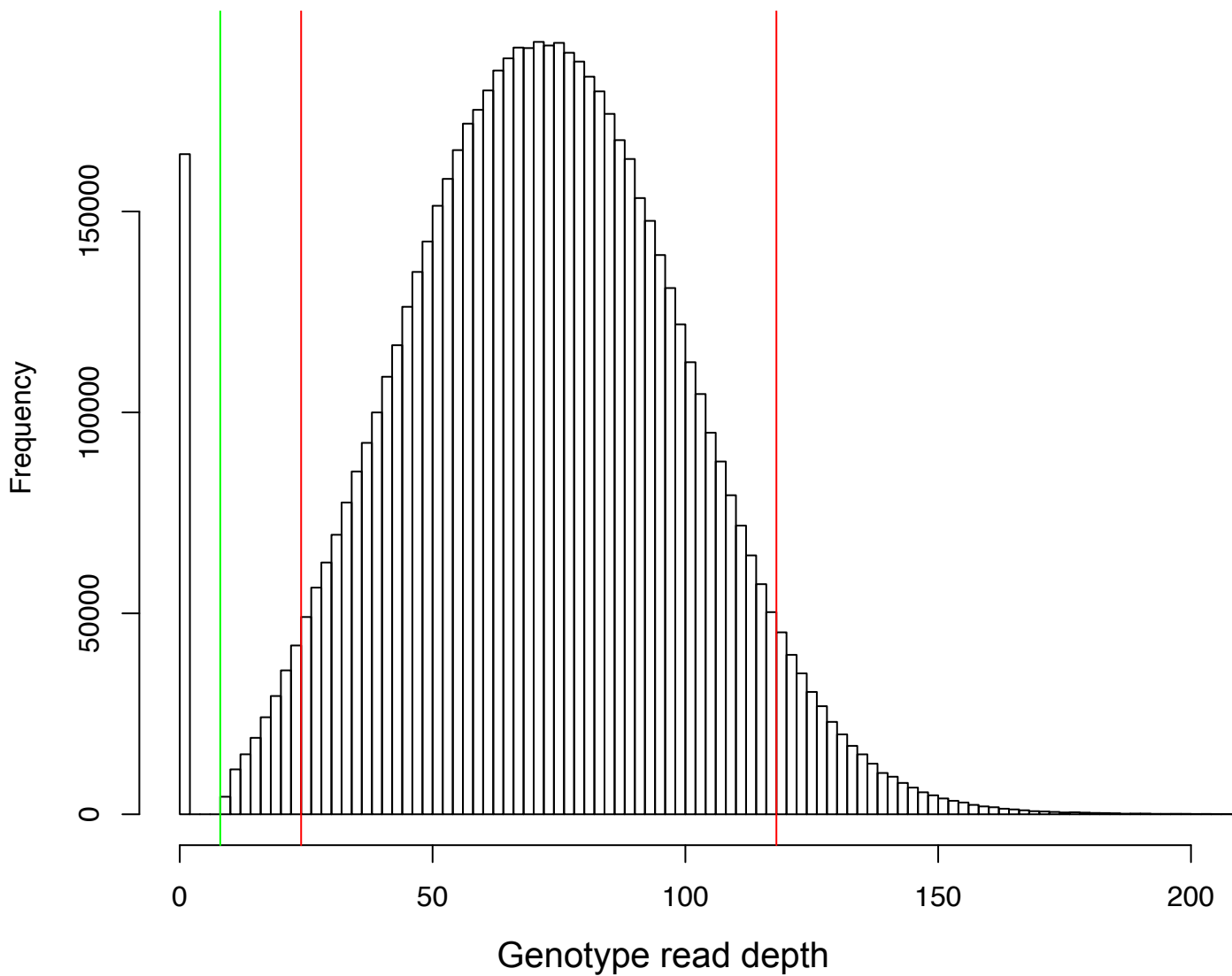

Supplement: Supplementary file 1 [file EVA-11-593-s001.pdf]

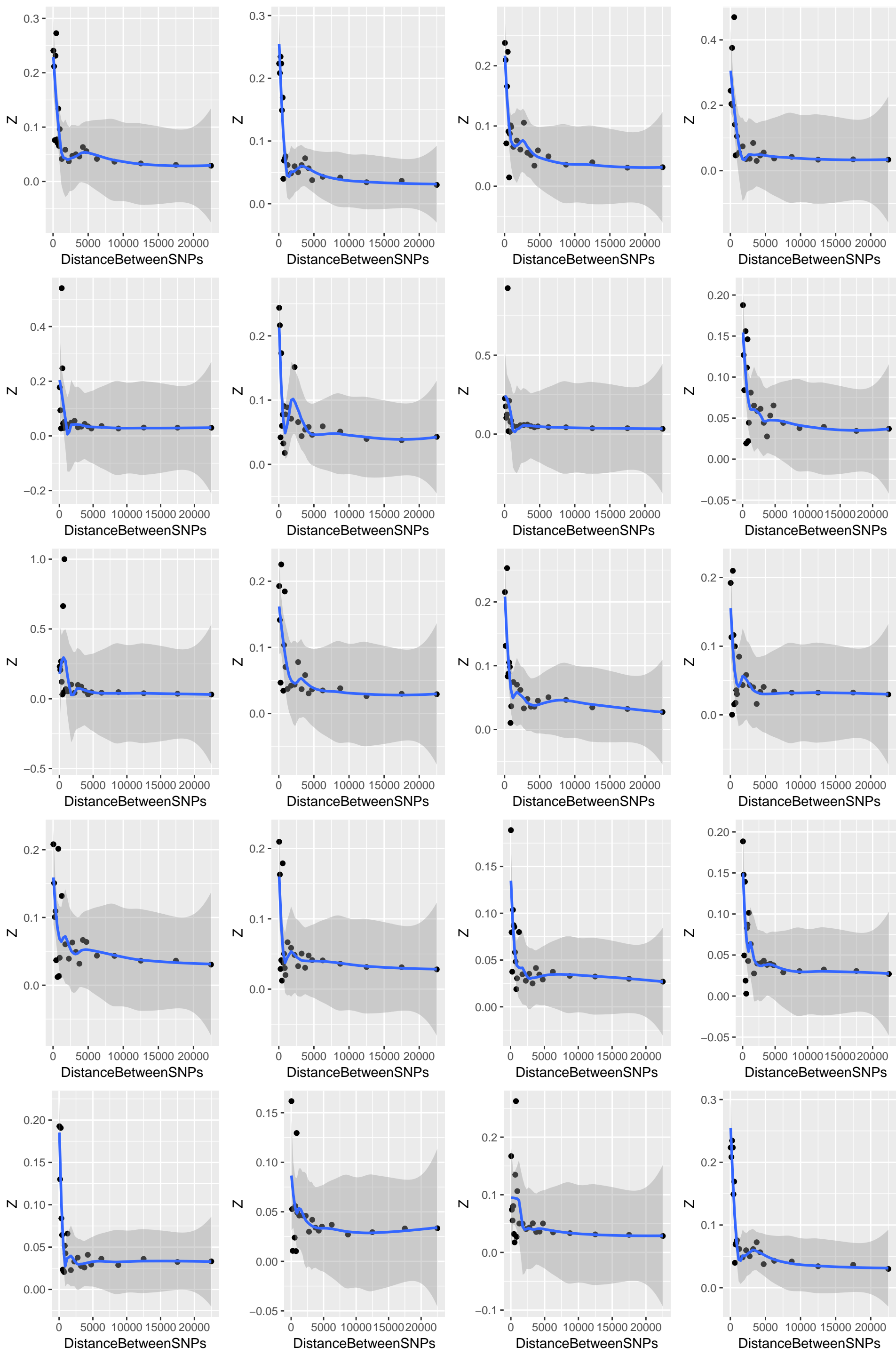

Supplement: Supplementary file 2 [file EVA-11-593-s002.pdf]

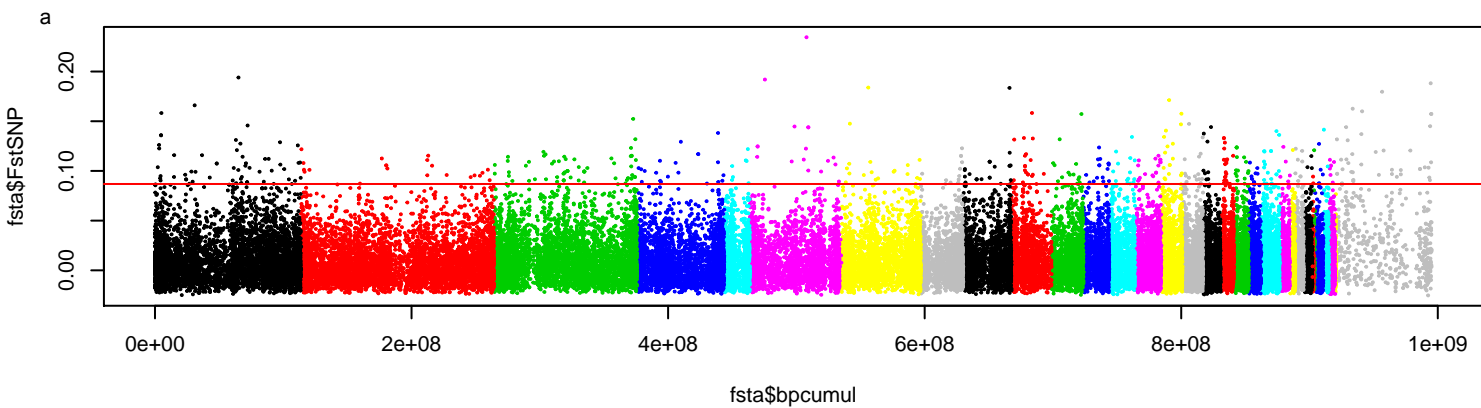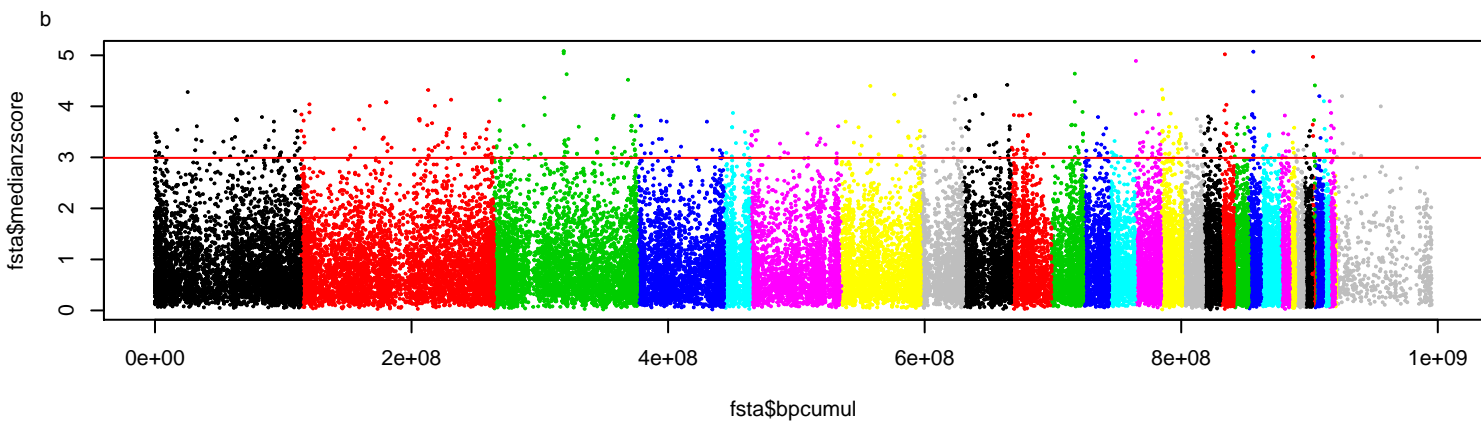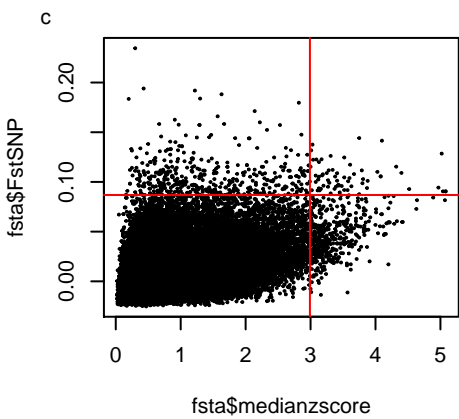

Supplement: Supplementary file 3 [file EVA-11-593-s003.pdf]

**A**

PCA cumulated  
eigenvalues

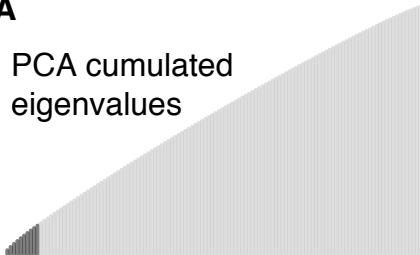

Ratio of correct assignment

**B**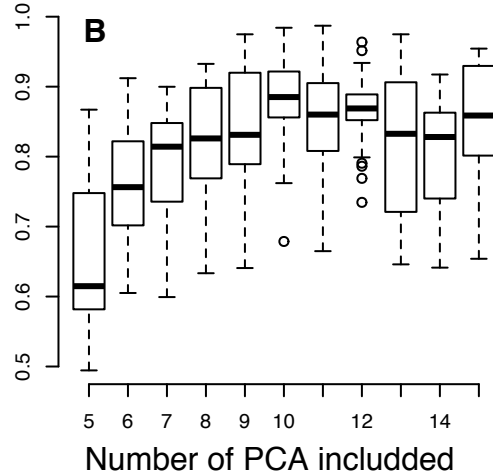**C**

DA eigenvalues

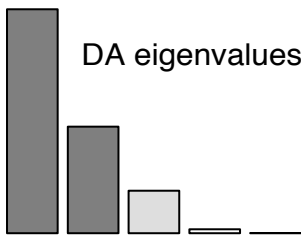**D**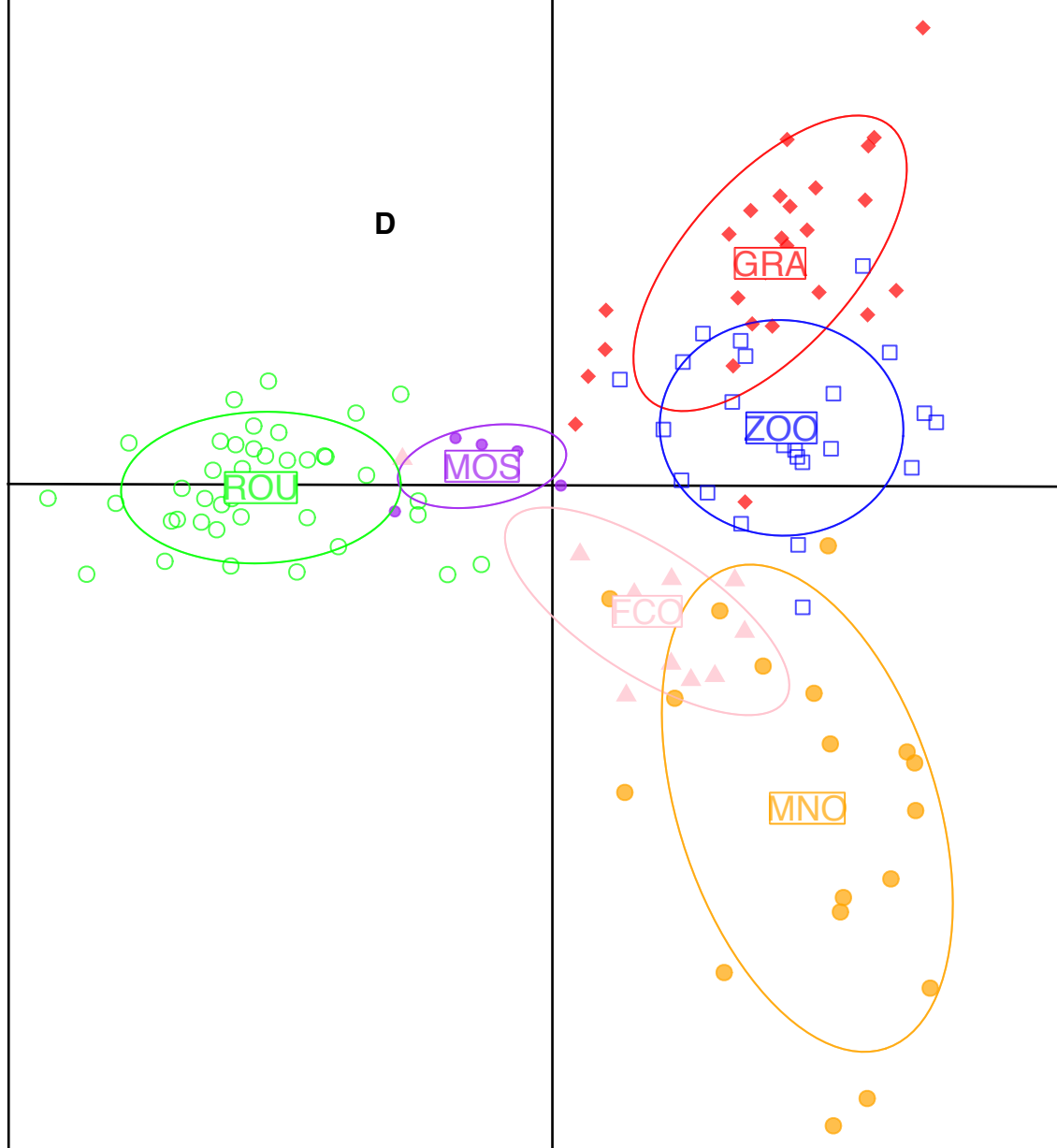

Supplement: Supplementary file 4 [file EVA-11-593-s004.pdf]

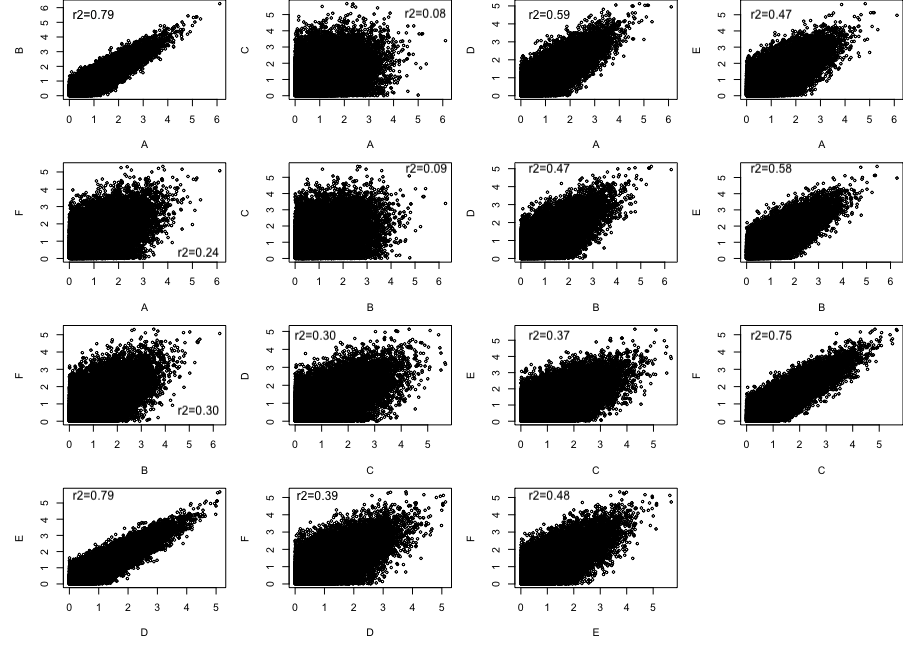

Supplement: Supplementary file 5 [file EVA-11-593-s005.png]

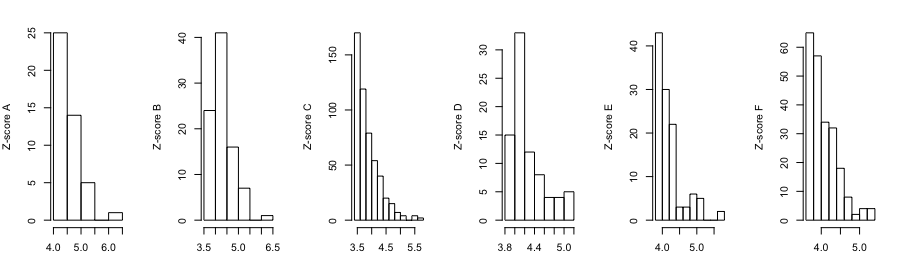

Supplement: Supplementary file 6 [file EVA-11-593-s006.png]

A- 3 tests with the zoo

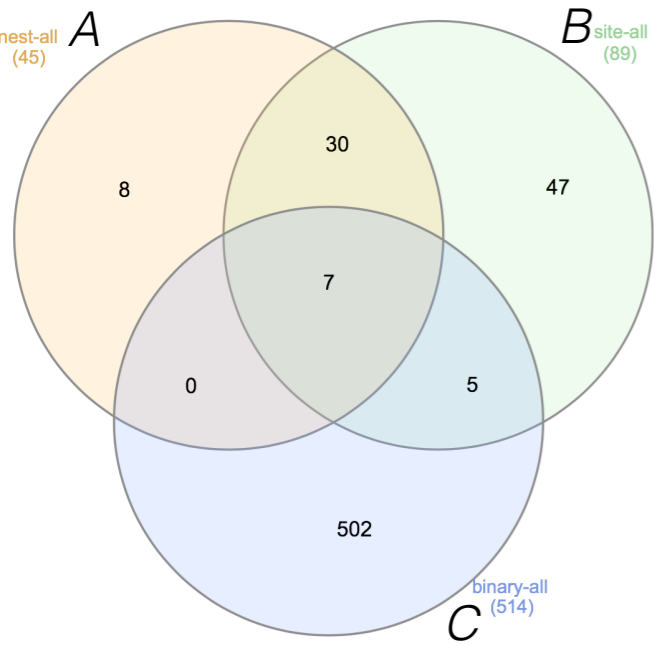

B- 3 tests without the zoo

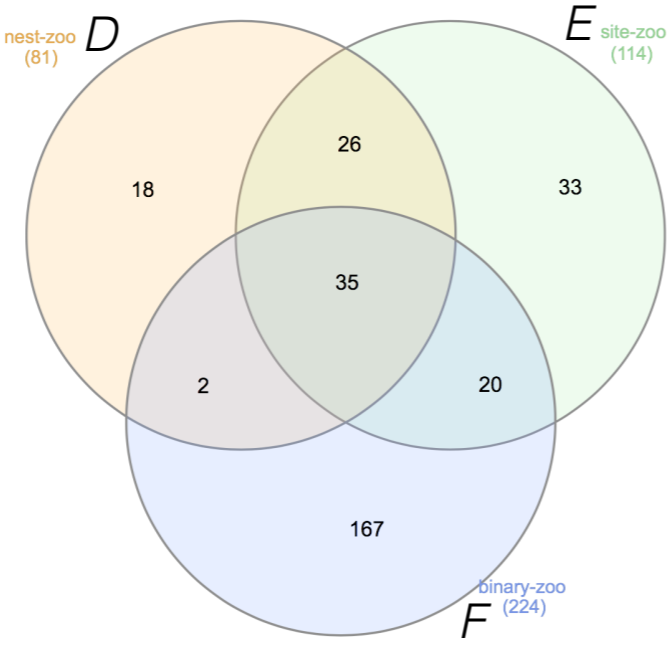

C- with vs without the zoo

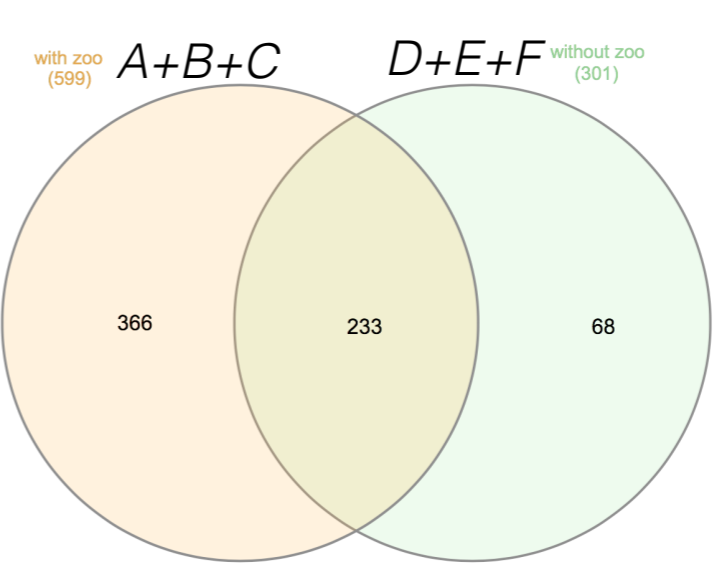

D- type of test

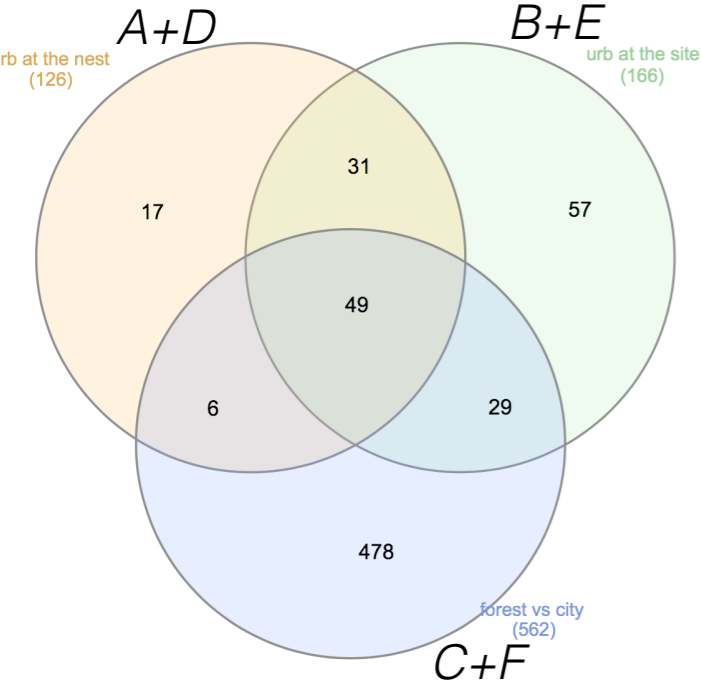

E- 6 tests

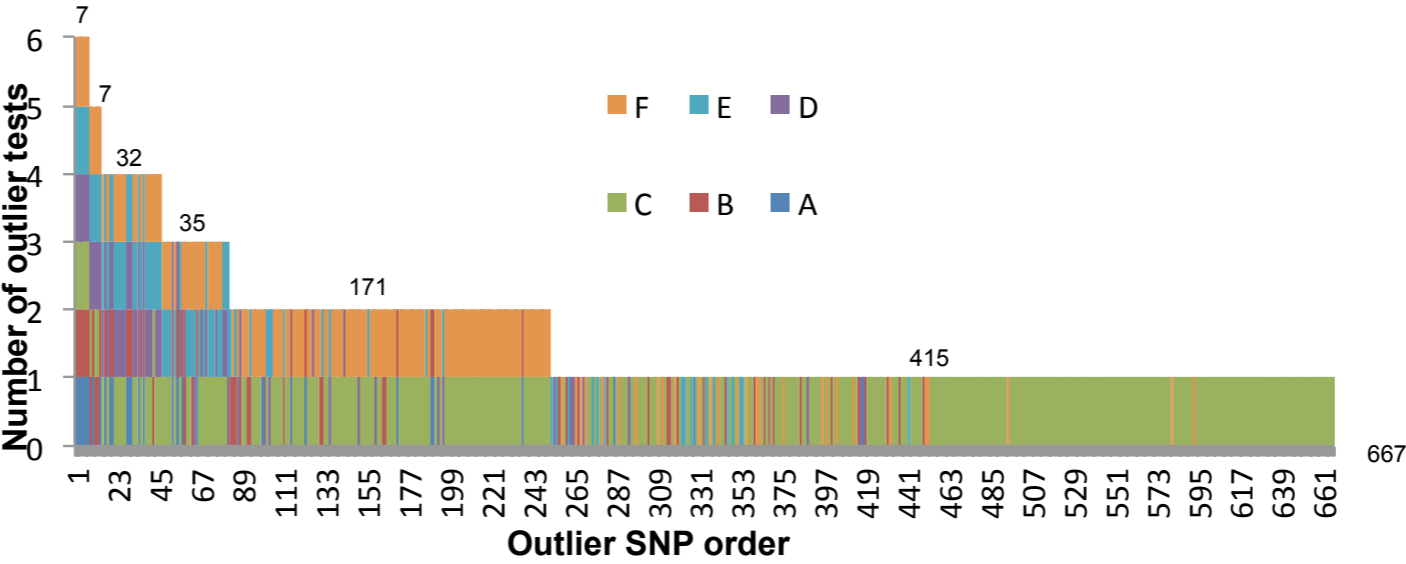

Supplement: Supplementary file 7 [file EVA-11-593-s007.pdf]
